# Neddylation contributes to CD4^+^ T cell-mediated protective immunity against blood-stage *Plasmodium* infection

**DOI:** 10.1371/journal.ppat.1007440

**Published:** 2018-11-21

**Authors:** Qianqian Cheng, Jian Liu, Yujun Pei, Yaolin Zhang, Dawang Zhou, Weiqing Pan, Jiyan Zhang

**Affiliations:** 1 Department of Molecular Immunology, Institute of Basic Medical Sciences, Beijing, China; 2 State Key Laboratory of Cellular Stress Biology, Innovation Center for Cell Signaling Network, School of Life Sciences, Xiamen University, Fujian, China; 3 Department of Tropical Infectious Diseases, Second Military Medical University, Shanghai, China; University of Massachusetts Medical School, UNITED STATES

## Abstract

CD4^+^ T cells play predominant roles in protective immunity against blood-stage *Plasmodium* infection, both for IFN-γ-dependent effector mechanisms and providing B cell helper signals. Neddylation, an ubiquitination-like process triggered by covalent conjugation of NEDD8 to specific targets, has emerged as a potential regulator of T cell activities to TCR engagement. However, its contribution to T cell-mediated immunity to blood-stage malaria remains unclear. Here using an experimental model induced by *Plasmodium yoelii* 17XNL, and conditional knockout mice with T cell-specific deficiency of crucial components of neddylation pathway, we demonstrate activation of neddylation in T cells during blood-stage *Plasmodium* infection is essential for parasite control and host survival. Mechanistically, we show that apart from promoting CD4^+^ T cell activation, proliferation, and development of protective T helper 1 (Th1) cell response as suggested previously, neddylation is also required for supporting CD4^+^ T cell survival, mainly through B-cell lymphoma-2 (Bcl-2) mediated suppression of the mitochondria-dependent apoptosis. Furthermore, we provide evidence that neddylation contributes to follicular helper T (Tfh) cell differentiation, probably via augmenting the ubiquitin ligase Itch activity and proteasomal degradation of FoxO1, thereby facilitating germinal center (GC) formation and parasite-specific antibody production. This study identifies neddylation as a positive regulator of anti-*Plasmodium* immunity and provides insight into an involvement of such pathway in host resistance to infectious diseases.

## Author summary

Malaria, which is caused by the intracellular parasite *Plasmodium*, remains a major infectious disease with significant morbidity and mortality annually. Better understanding of the molecular mechanisms involved in protective immunity against the pathogenic blood-stage *Plasmodium* will facilitate development of anti-malarial drugs and vaccines. Neddylation has recently been identified as a potential regulator of T cell function. Here, we directly addressed the effects of neddylation on T cell responses and the outcome of blood-stage *P*. *yoelii* 17XNL malaria. We show that activation of neddylation in T cells is essential for IFN-γ-mediated proinflammatory response and generation of parasite-specific antibodies, thus contributing to full resolution of the infection. This is primarily associated with the reported beneficial effects of neddylation on CD4^+^ T cell activities, including activation, proliferation, and differentiation into T helper 1 (Th1) cells. Additionally, we establish a novel role of neddylation in parasite-responsive CD4^+^ T cell survival and follicular helper T (Tfh) cell differentiation. Therefore, we provide evidence that neddylation may represent a novel mechanism in orchestrating optimum CD4^+^ T cell effector response and subsequent humoral immunity to blood-stage *Plasmodium* infection.

## Introduction

Malaria remains the most devastating parasitic diseases worldwide [1]. One major hurdle in eradicating malaria is attributed to the incomplete understanding of host-parasite interactions, especially in the asexual blood stage of *Plasmodium* infection, which is pathogenic and responsible for all the symptoms of disease. Therefore, further dissection of the immunological mechanisms underlying host resistance to the blood-stage parasite is a prerequisite for developing novel interventions against malaria.

It is well established that effective control of blood-stage malaria depends on both cell-mediated immune responses and antibody-dependent humoral immunity [2–4]. T lymphocytes play indispensable roles in these processes. Activation of T cells, particularly CD4^+^ T cells, is critical for IFN-γ-mediated proinflammatory response, thereby facilitating macrophage activation and phagocytosis of captured parasites during acute infection [5, 6]. Moreover, CD4^+^ T cells are crucial helpers to support B cell affinity maturation and generation of parasite-specific immunoglobulin G (IgG), thus contributing to complete elimination of the infection eventually [7–9]. As such, CD4^+^ T cells are key participants in protection against blood-stage *Plasmodium* infection, which makes them potential targets of the complex signaling networks regulating anti-malarial immunity. However, the molecular pathways that directly regulate CD4^+^ T cell activities or the interplay with B cells during malaria remain elusive.

Neddylation is a post-translational modification in which the ubiquitin-like modifier NEDD8 is covalently conjugated to substrate proteins [10]. Similar to ubiquitination, it is triggered by the successive action of NEDD8 activating enzyme E1 (NAE, heterodimer of APPBP1 and the catalytic subunit Uba3), NEDD8-conjugating enzyme E2 (Ubc12 or Ube2M) and NEDD8-E3 ligases [11]. The best characterized substrates of neddylation are cullins [11,12]. Our knowledge about the role of neddylation in various cellular processes depends largely on studies using MLN4924, a pharmacological inhibitor of NAE, which blocks NEDD8 activation and, consequently, the neddylation pathway [13]. Recently, an implication of protein neddylation in immunological regulation has been described. It has been shown that blockade of neddylation pathway either by MLN4924 treatment, or shRNA-mediated knockdown of Ubc12, could attenuate TCR-induced CD4^+^ T cell activation and proliferation, as well as differentiation into T helper 1 (Th1) and T helper 1 (Th2) cells under the corresponding skewing conditions. Moreover, neddylation is required for Th2-mediated allergic response in OVA-driven airway inflammation [14]. Therefore, neddylation may serve as a regulator of T cell functions. However, a role for this pathway in T cell-mediated immunity to infectious agents is largely unexplored.

In the present study, we investigated the functional relevance of neddylation with T cell responses, as well as association with the consequence of blood-stage malaria. Using an experimental model induced by infection with the non-lethal *P*. *yoelii* 17XNL strain, and mice with a genetic deletion of Uba3 or NEDD8 in T cells, we confirmed the significance of neddylation in promoting T cell-mediated optimum IFN-γ and anti-parasite IgG responses, and thus timely control of parasitemia and host survival during *Plasmodium* infection. This effect was closely associated with regulation of numerous aspects of CD4^+^ T cell activities, including proliferation, Th1 cell differentiation, B-cell lymphoma-2 (Bcl-2) supported CD4^+^ T cell survival, as well as follicular helper T (Tfh) cell differentiation, which was largely associated with Itch targeted proteasomal degradation of FoxO1. Therefore, we have described a previously unrecognized molecular pathway in regulating CD4^+^ T cell-mediated protective immunity to blood-stage malaria, and provided an insight into the involvement of neddylation in host resistance to infectious diseases.

## Results

### Blood-stage *P*. *yoelii* 17XNL infection activates neddylation in T cells

**Fig 1:**
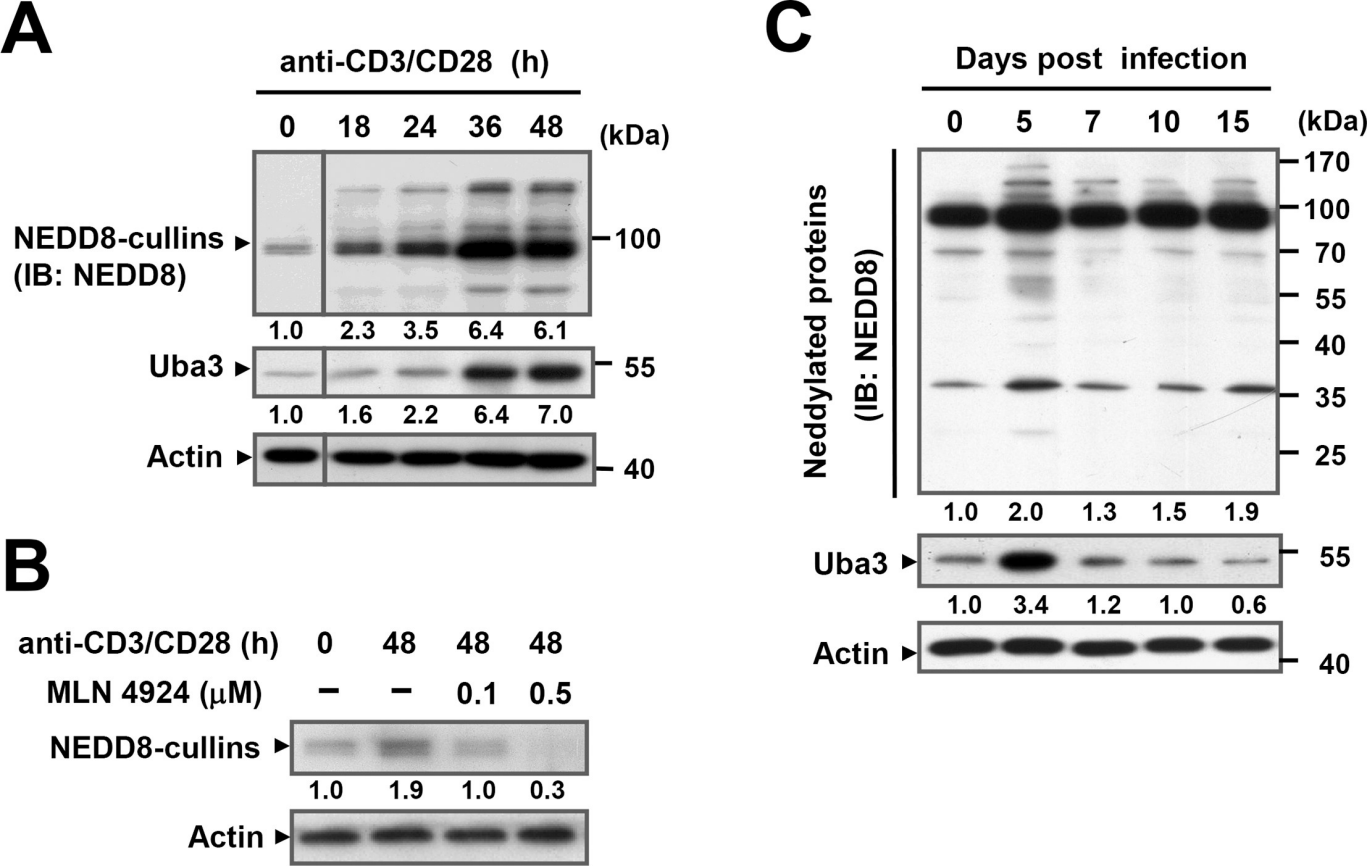
Activation of neddylation in T cells upon TCR ligation and blood-stage *P*. *yoelii* 17XNL infection. (A) Immunoblotting of total cell extracts from splenic T cells of C57BL/6 mice stimulated with CD3/CD28 Dynabeads for various times (different parts of the same gel were grouped together with a dividing line). Uba3 and NEDD8-conjugated proteins were detected using anti-Uba3 and anti-NEDD8 antibodies, respectively. (B) Splenic T cells purified from C57BL/6 mice were incubated with MLN4924 (0.1, 0.5 μM) or DMSO (–) for 1h, then left untreated or stimulated with CD3/CD28 Dynabeads for 48h, NEDD8-conjugated cullins were detected by probing with anti-NEDD8 antibody. (C) Immunoblotting for Uba3 and NEDD8-conjugated proteins in splenic T cells isolated from C57BL/6 mice at indicated time points of blood-stage *P*. *yoelii* 17XNL infection, Uba3 and neddylated proteins were detected by probing with anti-Uba3 and anti-NEDD8 antibodies. Numbers indicate relative density of the bands quantified by scanning densitometry, normalized to β-Actin and are presented relative to unstimulated cells (0 h) or uninfected mice (day 0). Data are representative of three independent experiments with similar results.

We firstly examined TCR-induced neddylation *in vitro*. For this, splenic T cells purified from C57BL/6 mice were stimulated with T-Activator CD3/CD28 Dynabeads, in the presence or absence of the well-established NAE inhibitor, MLN4924 [13]. Immunoblotting analysis revealed that TCR ligation led to a rapid induction of Uba3, which was accompanied by increased NEDD8-conjugated cullins (molecular weight: 90–100 kDa) [15] and other NEDD8-modified proteins (Fig 1A). As anticipated, MLN4924 treatment could efficiently inhibit this process in a dose-dependent manner (Fig 1B). Then we assessed the relationship between neddylation in antigen-primed T cells and blood-stage *Plasmodium* infection. To this end, C57BL/6 mice were inoculated intraperitoneally (i.p.) with 3×10^4^ parasitized erythrocytes of a self-resolving parasite strain, *P*. *yoelii* 17XNL. In parallel with the observations *in vitro*, T cells expressed dramatically increased amounts of Uba3 and NEDD8-conjugated proteins within 5 days of the infection (Fig 1C), confirming activation of neddylation in parasite-responsive T cells. Therefore, TCR stimuli could induce Uba3 up-regulation and NEDD8 modification of target proteins, and that *P*. *yoelii* 17XNL-driven neddylation might allude to a potential role for this pathway in T cell-mediated defense against blood-stage malaria.

### Neddylation in T cells is essential for host resistance to blood-stage *P*. *yoelii* 17XNL infection

**Fig 2:**
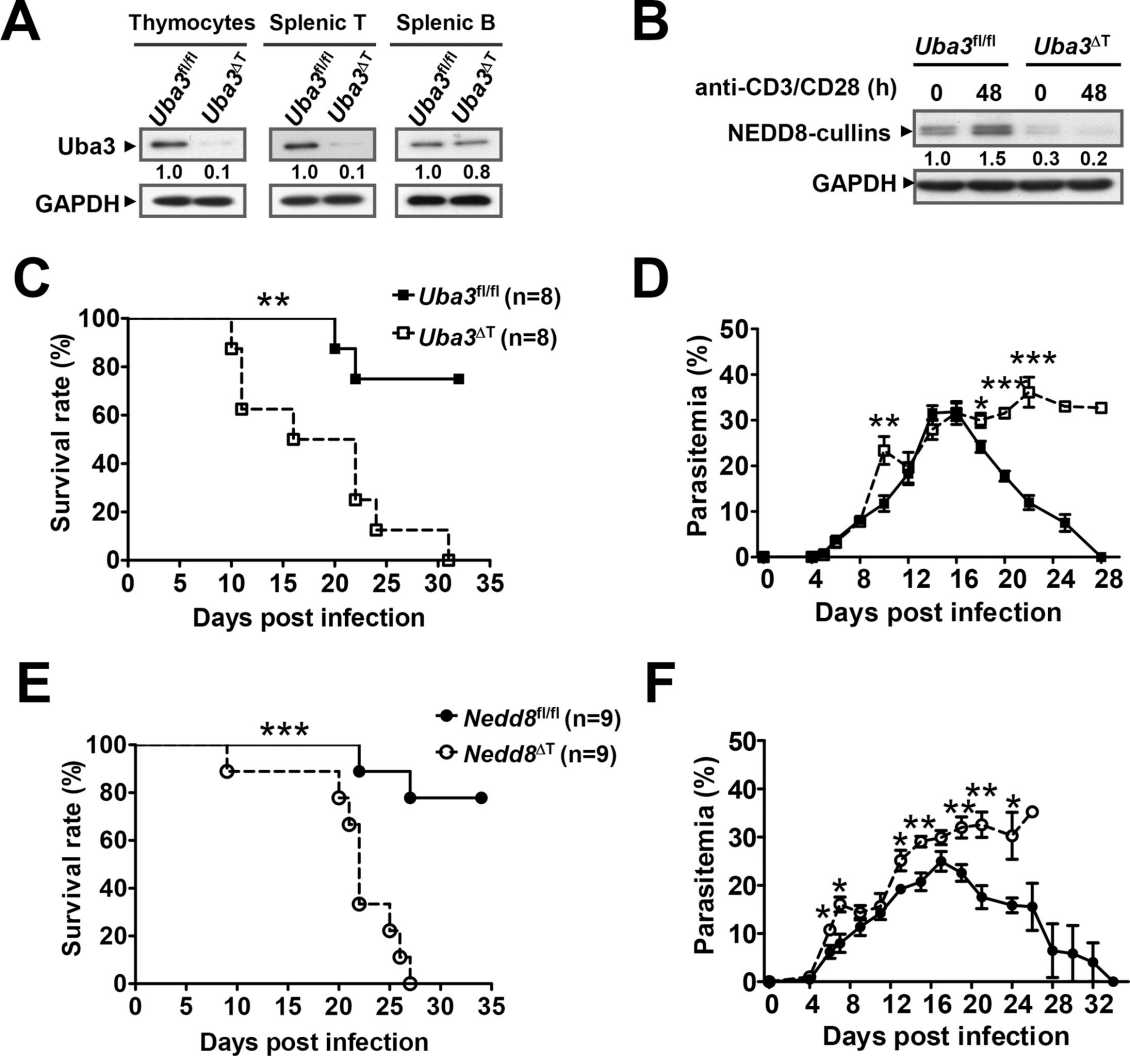
Neddylation in T cells contributes to resolution of blood-stage *P*. *yoelii* 17XNL infection. (A) Immunoblotting of total cell extracts from thymocytes, splenic T (CD3^+^) cells, or B (B220^+^) cells of *Uba3*^*fl/fl*^*Lck-Cre*^+^ (*Uba3*^ΔT^) and *Uba3*^*fl/fl*^ mice. T cell-specific Uba3 deficiency was validated by probing with anti-Uba3 antibody. Numbers indicate densitometry of the bands relative to that of *Uba3*^*fl/fl*^ mice. (B) Splenic T cells purified from *Uba3*^*fl/fl*^ and *Uba3*^ΔT^ mice were left either untreated or stimulated with CD3/CD28 Dynabeads for 48h, NEDD8-conjugated cullins was detected by probing with anti-NEDD8 antibody. Numbers indicate densitometry of the bands relative to that of unstimulated *Uba3*^*fl/fl*^ cells (0 h). (C-D) Groups of *Uba3*^*fl/fl*^ and *Uba3*^ΔT^ mice were infected i.p. with 3×10^4^ parasitized erythrocytes of *P*. *yoelii* 17XNL (n = 8 per group), (C) survival curves were analyzed by log-rank test, (D) parasitemia values are presented as mean±SEM and analyzed by Mann-Whitney U test. (E-F) Course of *P*. *yoelii* 17XNL infection in *Nedd8*^*fl/fl*^ and *Nedd8*^ΔT^ mice (n = 9 per group), overall survival and parasitemia were analyzed as described above. Data shown are from one of three independent experiments with similar results. * *p*<0.05, ** *p*<0.01, ***p<0.001.

To directly elucidate the effects of neddylation on T cell-mediated defense against *Plasmodium* infection, we crossed *Uba3*^*fl/fl*^ mice with the *Lck-Cre* transgeneic strain to generate *Uba3*^*fl/fl*^*Lck-Cre*^+^ (named as *Uba3*^ΔT^) mice, in which deletion of *Uba3* was confined to the T cell compartment (Fig 2A). As expected, Uba3 deficiency led to remarkably reduced NEDD8-conjugated cullins either before or after TCR stimulation in splenic T cells, confirming efficient blockade of neddylation in the mutants (Fig 2B). After challenging with 3×10^4^ parasitized erythrocytes of *P*. *yoelii* 17XNL, *Uba3*^*fl/fl*^ mice developed a peak parasitemia of approximately 30% at day 16 post infection (p.i.), most (75%) of them were able to eliminate the parasites and fully recover from the infection within 28 days, whereas *Uba3*^ΔT^ mice suffered from unremitting hyperparasitemia and all succumbed to the infection by day 31 (*p*<0.01 for survival comparison) (Fig 2C and 2D), suggesting that Uba3 deficiency in T cells significantly increased the susceptibility of mice to *P*. *yoelii* 17XNL infection. Owing to the pivotal role for Uba3 in initiating the neddylation process, it is reasonable to speculate that neddylation in T cells may contribute to the resolution of infection.

To substantiate this hypothesis, we performed *P*. *yoelii* 17XNL infection in another conditional knockout model with a T cell-specific deficiency of NEDD8 (*Nedd8*^*fl/fl*^*Lck-Cre*^+^, named as *Nedd8*^ΔT^ mice) (S1 Fig). As predicted, *Nedd8*^ΔT^ mice exhibited greater parasite burdens and 100% mortality within 27 days (*p*<0.01 for survival compared to *Nedd8*^*fl/fl*^ littermate controls) (Fig 2E and 2F), which closely resembled the course of infection in *Uba3*^ΔT^ mice. Together, these data support the idea that neddylation in T cells is critically required for host resistance to blood-stage *Plasmodium* infection.

### Neddylation in T cells promotes optimum IFN-γ and humoral immune responses during *P*. *yoelii* 17XNL infection

**Fig 3:**
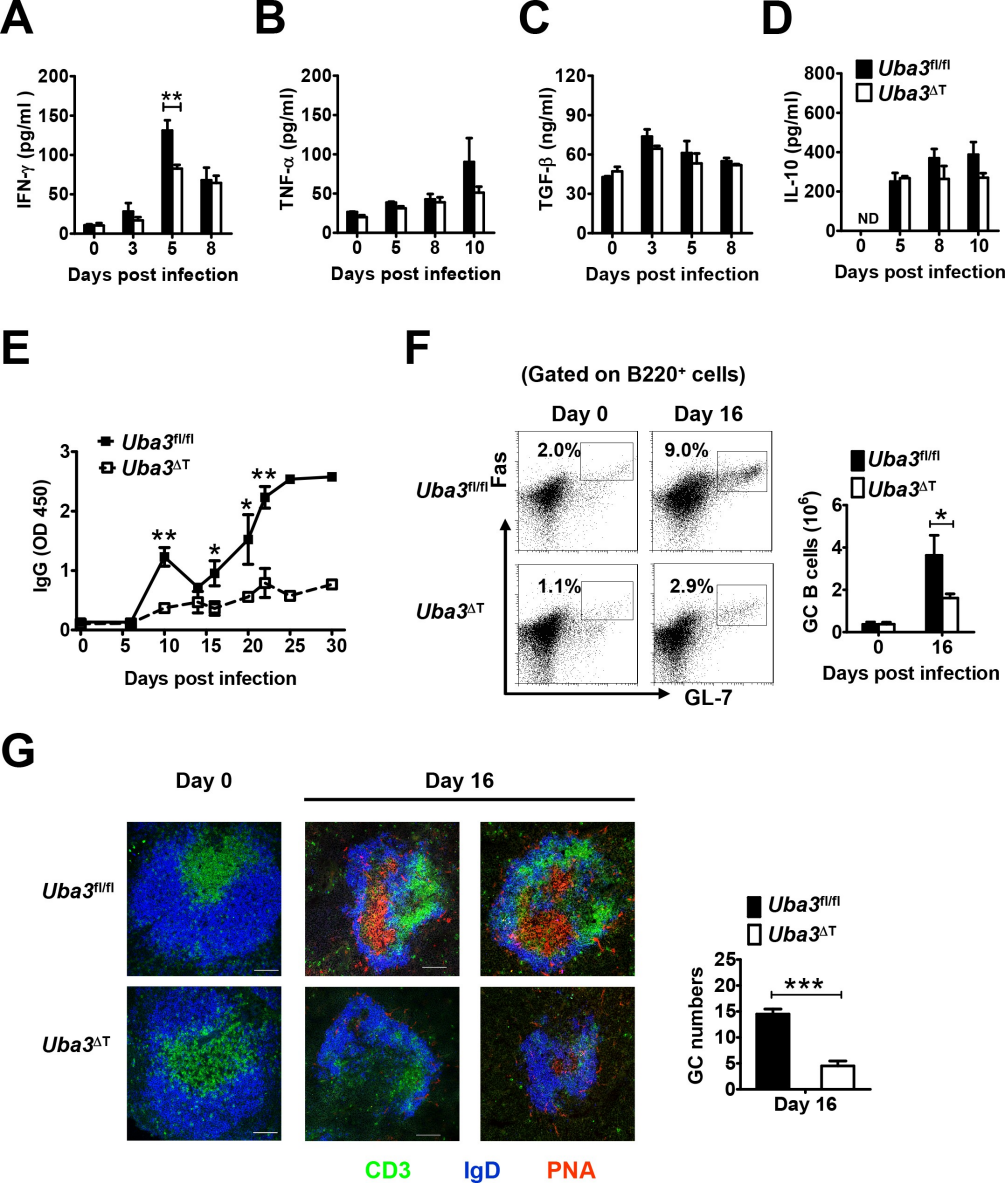
Defective IFN-γ and anti-*P*. *yoelii* 17XNL humoral immune responses in *Uba3*^ΔT^ mice. Serum levels of proinflammatory cytokines (A) IFN-γ, (B) TNF-α, and anti-inflammatory cytokines (C) TGF-β, (D) IL-10 in *Uba3*^*fl/fl*^ and *Uba3*^ΔT^ mice at days 0, 3, 5, 8, 10 of *P*. *yoelii* 17XNL infection (n = 5–6 per group), as determined by ELISA. (E) Parasite-specific IgG response over the course of infection was determined by ELISA. Data are expressed as relative OD values (n = 6 per group). (F) Representative dot plots and bar graphs showing the proportions and absolute numbers of Fas^+^GL-7^+^ germinal center B cells (gated on live B220^+^ B cells) in spleens of *Uba3*^*fl/fl*^ and *Uba3*^ΔT^ mice at day 0 and day 16 p.i. (n = 5–6 per group). (G) Left, representative confocal micrographs of spleen sections from day 0 and day16 infected *Uba3*^*fl/fl*^ and *Uba3*^ΔT^ mice, identifying T cell zones with anti-CD3 (green), B cell follicles with anti-IgD (blue), and germinal centers (GCs) with peanut agglutinin (PNA, red) staining. Scale bars, 100 μm. Right, bar graphs showing GC numbers per two sections from the spleen of each mouse at day 16 p.i. (n = 3 per group). Data are representative of three replicate experiments and are shown as mean±SEM. *p<0.05, ** *p*<0.01, ***p<0.001 by Student’s *t* test.

**Fig 4:**
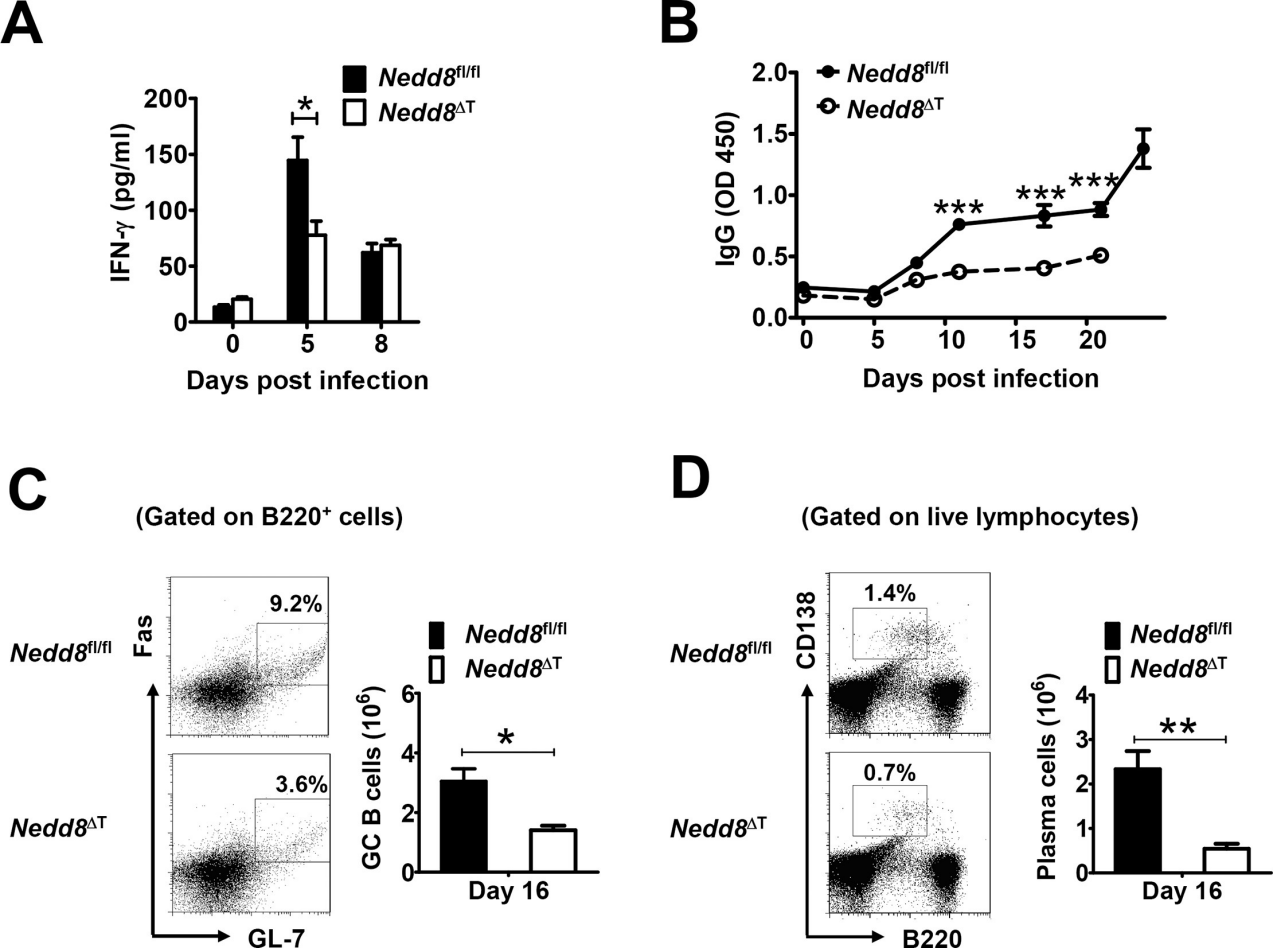
Defective IFN-γ and anti-*P*. *yoelii* 17XNL humoral immune responses in *Nedd8*^ΔT^ mice. (A) Serum levels of IFN-γ in *Nedd8*^*fl/fl*^ and *Nedd8*^ΔT^ mice at days 0, 5, 8 p.i. (n = 4–5 per group). (B) Serum levels of anti-*P*. *yoelii* 17XNL IgG in *Nedd8*^*fl/fl*^ and *Nedd8*^ΔT^ mice during the course of infection (n = 5 per group). (C-D) Representative dot plots and bar graphs showing the proportions and total numbers of (C) GL-7^+^Fas^+^ GC B cells (gated on live B220^+^ B cells), and (D) CD138^+^B220^lo^ plasma cells (gated on live lymphocytes) in spleens of *Nedd8*^*fl/fl*^ and *Nedd8*^ΔT^ mice at day 16 p.i. (n = 4–5 per group). Data are representative of three replicate experiments and are shown as mean±SEM. *p<0.05, ** *p*<0.01, ***p<0.001 by Student’s *t* test.

To explore how neddylation promotes T cell-mediated immunity to *P*. *yoelii* 17XNL, we focused on the immune mediators that are essential for resolution of blood-stage malaria. Given that optimal induction of proinflammatory cytokines (e.g., IFN-γ and TNF-α), and the counterbalance exerted by anti-inflammatory cytokines (TGF-β and IL-10) are crucial for determining the outcome of blood-stage malaria [16–19], we first analyzed the serum levels of these molecules. Notably, there was a remarkable elevation of IFN-γ in *Uba3*^*fl/fl*^ mice early after *P*. *yoelii* 17XNL infection, with the peak level significantly higher than that in *Uba3*^ΔT^ mice at day 5 p.i. (Fig 3A). However, no apparent differences were observed in serum TNF-α, TGF-β, and IL-10 levels between the two groups (Fig 3B–3D). These data, together with the observation of reduced serum IFN-γ in *Nedd8*^ΔT^ mice compared to *Nedd8* ^*fl/fl*^ controls at day 5 p.i. (Fig 4A), clearly demonstrate that disruption of neddylation in T cells impairs IFN-γ-mediated proinflammatory response during the early phase of *P*. *yoelii* 17XNL infection.

Additionally, we prepared whole blood-stage *P*. *yoelii* 17XNL antigen and measured parasite-specific IgG by ELISA. Coincide with gradually declined parasitemia, a robust increase of anti-*P*. *yoelii* 17XNL IgG was found in the serum of *Uba3*^*fl/fl*^ mice from day 16 p.i. onward. In marked contrast, *P*. *yoelii* 17XNL-infected *Uba3*^ΔT^ mice exhibited only slightly elevated IgG over the entire course (Fig 3E). Given that germinal centers (GCs) within B cell follicles are fundamental for thymus (T)-dependent B cell activation and antibody production [20, 21], we consequently sought to investigate whether abrogating of neddylation in T cells affect GC formation. As anticipated, there was a 2-fold decrease in the frequency of GL-7^+^Fas^+^ GC B cells in the spleen of *Uba3*^ΔT^ mice at day 16 p.i., and the total number of GC B cells was reduced to ~ 45% of that in the *Uba3*^*fl/fl*^ controls (Fig 3F). Immunofluorescence staining of the spleen further revealed that, compared to the robust abundance of typical Peanut Agglutinin positive (PNA^+^) GCs in *Uba3*^*fl/fl*^ littermate controls at day 16 p.i., *Uba3*^ΔT^ mice developed significantly reduced numbers of PNA^+^ GCs accompanied with severe disruption of their architecture in B cell follicles (IgD staining) and loss of T cells (Fig 3G), which suggest substantially impaired T cell-dependent B cell responses in these mice. Then we examined further to confirm the phenotype in *Nedd8*^ΔT^ mice. As expected, a fairly similar defect in parasite-specific total IgG production and GC B cell formation was found in *P*. *yoelii* 17XNL-infected *Nedd8*^ΔT^ mice, as compared to *Nedd8* ^*fl/fl*^ littermates (Fig 4B and 4C). Moreover, the frequency and total number of plasma cells (defined as CD138^+^B220^lo^ lymphocytes) were much lower in the spleen of infected *Nedd8*^ΔT^ mice at day 16 p.i. (Fig 4D). Therefore, disruption of neddylation in T cells also severely impairs normal GC reaction, and impedes establishment of anti-*P*. *yoelii* 17XNL humoral immune responses.

As a composite, these findings highlight the necessity of neddylation in the development of optimum IFN-γ response and protective humoral immunity to *P*. *yoelii* 17XNL infection, thus implying the integral role for this pathway in both the effector and helper function of T cells during blood-stage malaria.

### Neddylation in CD4^+^ T cells plays a prominent role in T cell-mediated immunity to *P*. *yoelii* 17XNL

**Fig 5:**
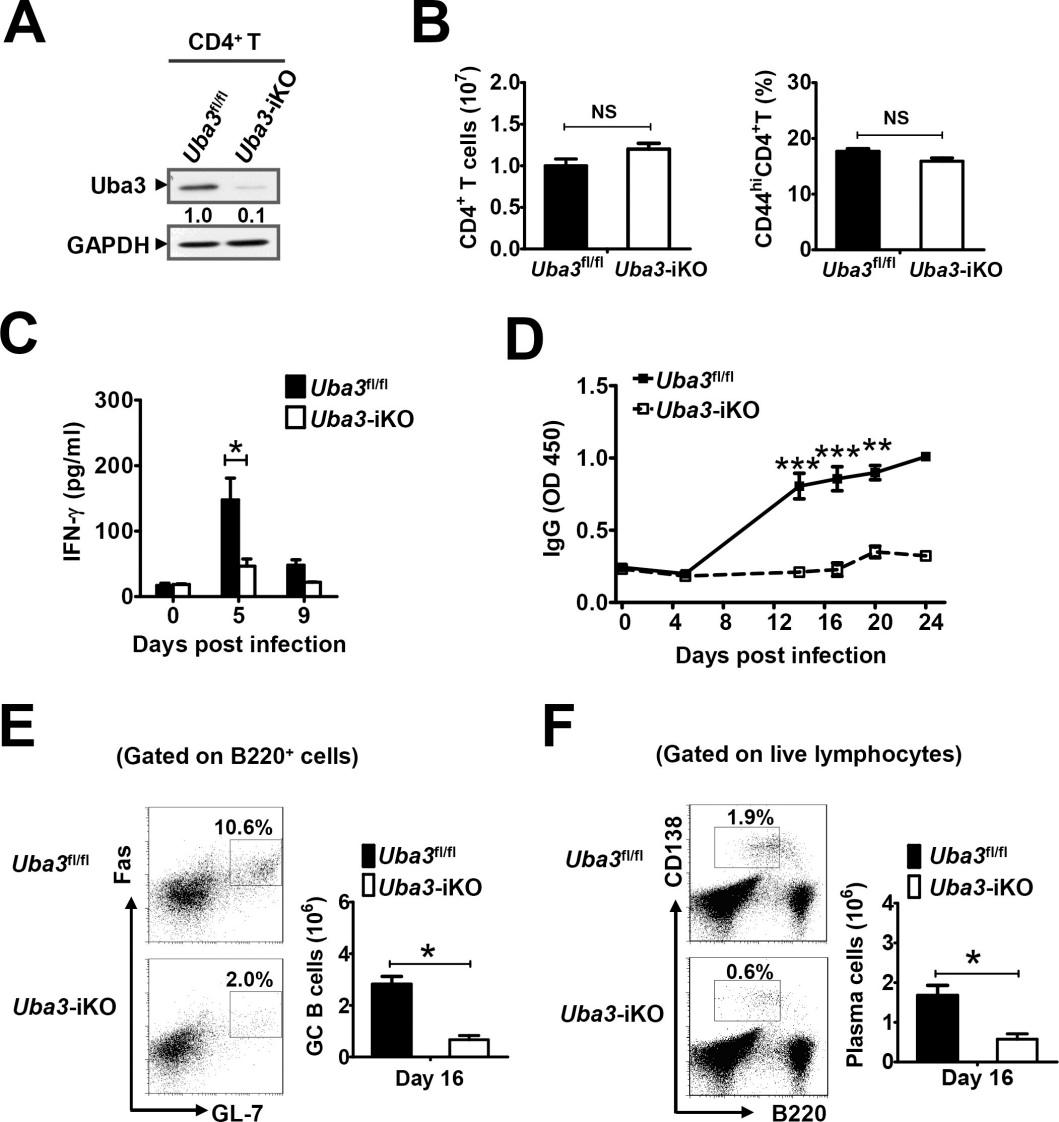
Neddylation in CD4^+^ T cells plays a crucial role in anti-*P*. *yoelii* 17XNL immunity. (A) Validation of Uba3 expression in splenic CD4^+^ T cells from tamoxifen-treated *Uba3*^*fl/fl*^ and *Uba3*^*fl/fl*^*Cd4-Cre*ER^T2^ (*Uba3*-iKO) mice. Numbers indicate densitometry of the bands relative to that of *Uba3*^*fl/fl*^ mice. (B) Numbers of total CD4^+^ T cells and activated CD44^hi^CD4^+^ T cells in spleens of *Uba3*^*fl/fl*^ and *Uba3*-iKO mice prior to *P*. *yoelii* 17XNL infection (n = 4 per group). (C) Serum levels of IFN-γ in *Uba3*^*fl/fl*^ and *Uba3*-iKO mice at days 0, 5, 9 p.i. (n = 5–6 per group). (D) Serum levels of anti-*P*. *yoelii* 17XNL IgG in *Uba3*^*fl/fl*^ and *Uba3*-iKO mice during the course of infection (n = 5–6 per group). (E-F) Representative dot plots and bar graphs showing the proportions and total numbers of (E) GC B cells, and (F) plasma cells in spleens of *Uba3*^*fl/fl*^ and *Uba3*-iKO mice at day 16 p.i. (n = 4 per group). Data are representative of three replicate experiments and expressed as mean±SEM. *p<0.05, ** *p*<0.01, and ***p<0.001 by Student’s *t* test.

Considering the importance of CD4^+^ T cells in IFN-γ response, GC formation and parasite-specific antibody production, we asked whether the impaired anti-*P*. *yoelii* 17XNL immunity in *Uba3*^ΔT^ mice was primarily dependent on neddylation pathway in CD4^+^ T cells. To address this, we crossed *Uba3*^*fl/fl*^ mice with *Cd4-CreER*^*T2*^ transgeneic mice (expressing a tamoxifen-responsive Cre recombinase under the control of *Cd4* promoter) to generate the *Uba3*^*fl/fl*^*Cd4-CreER*^*T2*^ model, which enabled us to inducibly and specifically delete *Uba3* in peripheral CD4^+^ T cells [22]. Immunoblotting analysis confirmed that Uba3 expression was almost completely eliminated in splenic CD4^+^ T cells after i.p. injection with 2 mg tamoxifen daily for consecutive 5 days (referred to as *Uba3*-iKO mice thereafter) (Fig 5A). Then these *Uba3*-iKO mice and their *Uba3*^*fl/fl*^ counterparts were infected with *P*.*yoelii* 17XNL on the following day. As expected, acute deletion of *Uba3* did not affect the initial number and activation status (CD44^hi^) of splenic CD4^+^ T cells prior to parasite infection (Fig 5B), but led to impaired production of serum IFN-γ (Fig 5C), parasite-specific IgG responses (Fig 5D), splenic GC B cell formation (Fig 5E), and plasma cell differentiation (Fig 5F), which closely mimicked the defective anti-*P*.*yoelii* 17XNL immunity seen in infected *Uba3*^ΔT^ and *Nedd8*^ΔT^ mice (Fig 3 and Fig 4). These data support the view that neddylation in CD4^+^ T cells plays a prominent role in IFN-γ and humoral immune responses during *P*. *yoelii* 17XNL infection.

### Neddylation promotes CD4^+^ T cell expansion and Th1 cell differentiation during *P*. *yoelii* 17XNL infection

**Fig 6:**
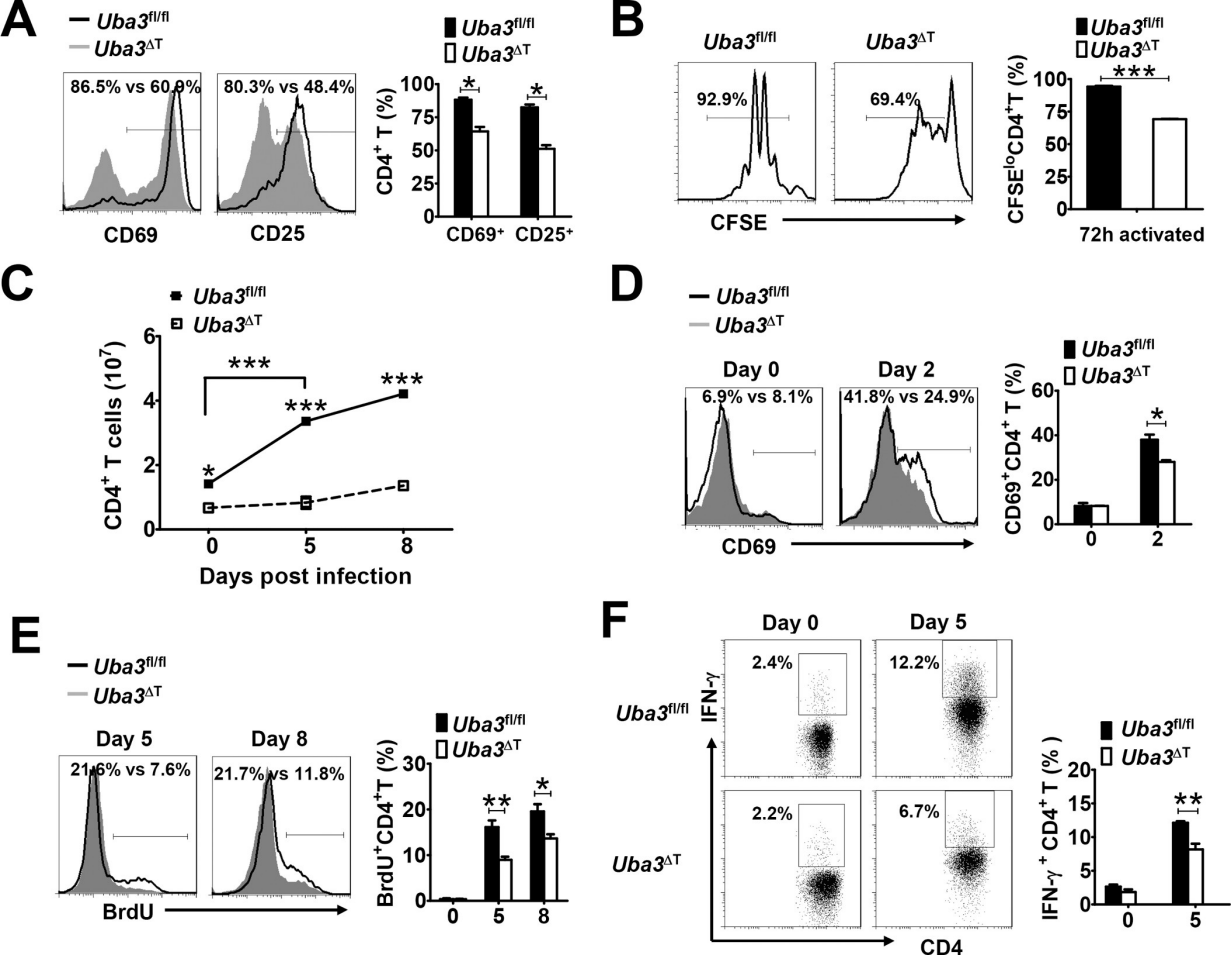
Neddylation promotes CD4^+^ T cell expansion and Th1 cell differentiation during *P*. *yoelii* 17XNL infection. (A) Representative histograms and bar graphs showing CD69 and CD25 expression on splenic CD4^+^ T cells from *Uba3*^*fl/fl*^ (black line) and *Uba3*^ΔT^ mice (gray-filled area) stimulated with CD3/CD28 Dynabeads for 10h. (B) CFSE labeled naive CD4^+^ T cells were stimulated with CD3/CD28 Dynabeads for 72h, cell proliferation was detected as CFSE dilution by flow cytometry. Data represent 3 mice per group from three independent experiments. *p<0.05, ***p<0.001 by Student’s *t* test. (C) Kinetics of splenic CD4^+^ T cell expansion in *Uba3*^*fl/fl*^ and *Uba3*^ΔT^ mice during the early phase of *P*. *yoelii* 17XNL infection (n = 5–6 per group). (D) Representative histograms and bar graphs showing CD69 expression on gated splenic CD4^+^ T cells from *Uba3*^*fl/fl*^ (black line) and *Uba3*^ΔT^ mice (gray-filled area) at day 0 and day 2 p.i. (n = 5 per group). (E) *P*. *yoelii* 17XNL-driven proliferation of splenic CD4^+^ T cells was detected by BrdU incorporation and flow cytometry. Representative histograms and bar graphs showing the proportions of proliferative (BrdU^+^) CD4^+^ T cells at days 0, 5, 8 p.i. (n = 5–6 per group). (F) Representative dot plots and summary graphs showing IFN-γ production by splenic CD4^+^ T cells at day 0 and day 5 p.i., as detected by intracellular staining (n = 5–6 per group). Data are representative of three replicate experiments and are shown as mean±SEM. *p<0.05, **p<0.01, ***p<0.01 by Student’s *t* test.

Our aforementioned data suggest that the beneficial effects of neddylation on T cell-mediated immunity to *P*. *yoelii* 17XNL are strongly dependent on CD4^+^ T cells. Therefore, we focused on this cell subset and evaluated whether Uba3 deficiency affects their activities, as suggested by Ubc12 knockdown previously [14]. As expected, up-regulation of the early activation molecules (e.g., CD69 and CD25) was relatively diminished on Uba3-defecient CD4^+^ T cells in response to CD3/CD28 stimuli (Fig 6A). In addition, Uba3-defecient CD4^+^ T cells exhibited greatly lower proliferative capacity than Uba3-sufficient CD4^+^ T cells, as determined by assessing Carboxyfluorescein succinmidyl ester (CFSE) dilution after 72h of culture (Fig 6B), which clearly demonstrated that blockade of neddylation lead to impaired functionalities of CD4^+^ T cells to TCR engagement.

Next, we investigated whether such alterations affect *P*. *yoelii* 17XNL-primed CD4^+^ T cell responses. As shown in Fig 6C, genetic deletion of Uba3 in T cells resulted in a slight reduction of CD4^+^ T cells in the spleen, and more notably, the difference was exaggerated after *P*. *yoelii* 17XNL infection. While *Uba3*^*fl/fl*^ mice displayed a 2.6-fold and 3.3-fold increase in total CD4^+^ T cells at day 5 and day 8 p.i., only a 1.7-fold increase was found in *Uba3*^ΔT^ mice at day 8 p.i. (Fig 6C). Therefore, neddylation is involved in rapid expansion of *P*. *yoelii* 17XNL-responsive CD4^+^ T cells. Further investigation on the functional capacity of splenic CD4^+^ T cells revealed that, Uba3-deficient CD4^+^ T cells displayed remarkably reduced CD69 expression at day 2 p.i. (Fig 6D), and significantly reduced proliferative response at day 5 and day 8 p.i., as measured by bromodeoxyuridine (BrdU) incorporation into the DNA of dividing cells (Fig 6E), confirming a requirement of neddylation in the initial activation and proliferation of *P*. *yoelii* 17XNL-primed CD4^+^ T cells. Additionally, intracellular cytokine staining revealed much lower frequency of IFN-γ-producing Th1 cells in *Uba3*^ΔT^ mice compared to that of *Uba3*^*fl/fl*^ mice at day 5 p.i. (Fig 6F), a similar trend in IL-2 production was also observed in CD4^+^ T cells (S2A Fig), whereas the proportions of IL-4-secreting Th2, IL-17A-secreting Th17 and regulatory T (Treg) cell subsets were not significantly affected (S2B–S2D Fig). Therefore, impaired serum IFN-γ response should be attributed to CD4^+^ T cells. Consistent with this notion, the proportions of IFN-γ-producing CD8^+^ T cells and γδ T cells were not significantly affected (S3A and S3B Fig), even though the absolute number of IFN-γ^+^ CD8^+^ T cells appeared to be lower in *Uba3*^ΔT^ spleens due to the reduced number of total CD8^+^ T cells (S3C and S3D Fig). Collectively, these data provide *in vivo* evidence that neddylation contributes to CD4^+^ T cell activation, proliferation, and development of optimum Th1 effector response that correlates with better protection against the early acute phase of blood-stage *P*. *yoelii* 17XNL infection.

### Neddylation improves *P*. *yoelii* 17XNL-responsive CD4^+^ T cell survival via the mitochondria-dependent pathway

**Fig 7:**
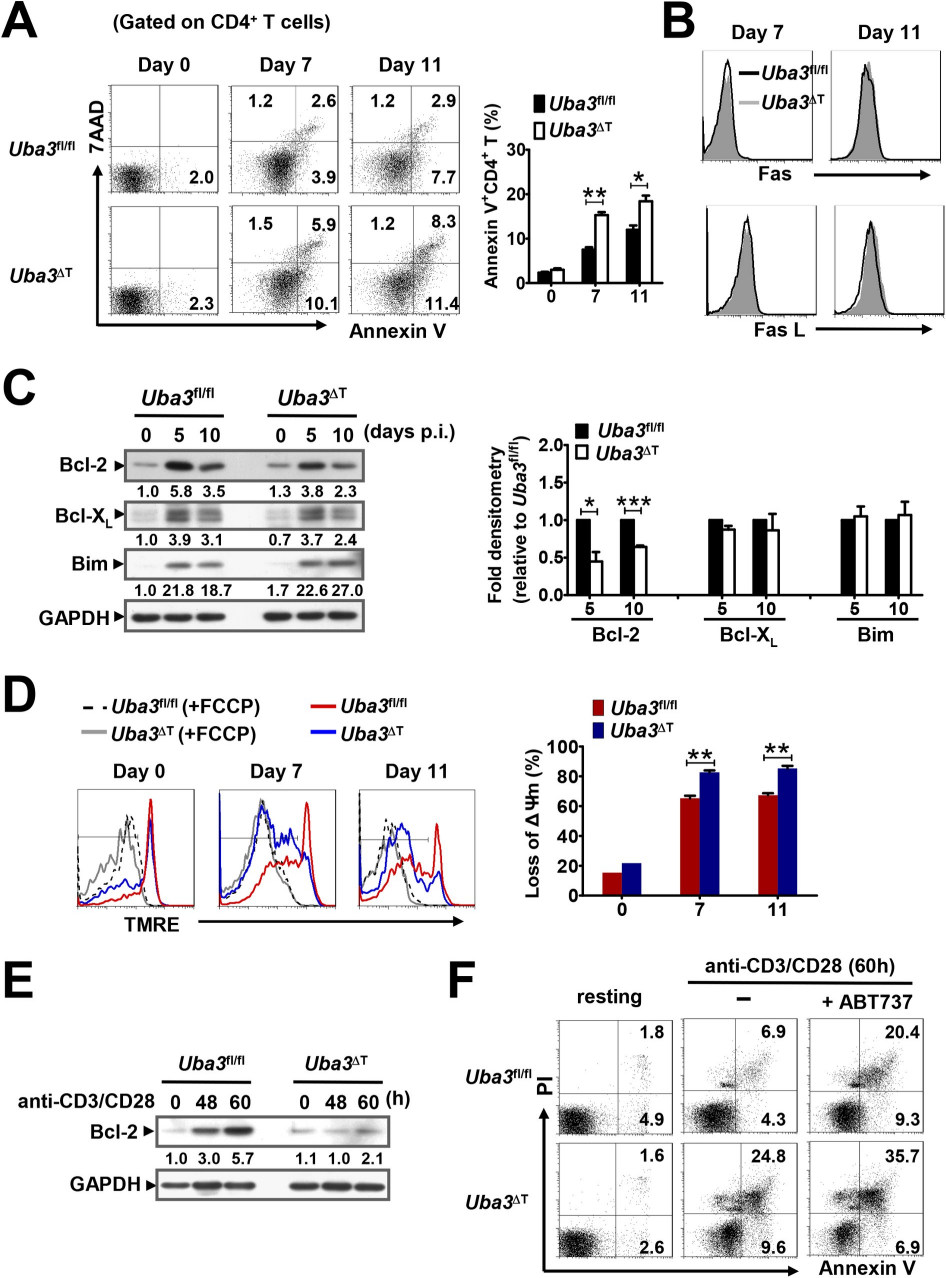
Neddylation improves CD4^+^ T cell survival via a mitochondria-dependent mechanism. (A) Representative dot plots showing Annexin V and 7AAD staining of splenic CD4^+^ T cells from *Uba3*^*fl/fl*^ and *Uba3*^ΔT^ mice at days 0, 7 and 11 p.i., bar graphs showing the proportions of total apoptotic Annexin V^+^ cells in the gated CD4^+^ T cell population (n = 6 per group). (B) Representative histograms showing Fas and FasL expression on splenic CD4^+^ T cells from *Uba3*^*fl/fl*^ (black line) and *Uba3*^ΔT^ mice (gray-filled area) at days 7, 11 p.i., data represent 3 mice per group from three independent experiments. (C) Left, representative immunoblots of Bcl-2, Bcl-X_L_ and Bim in splenic CD4^+^ T cells from *Uba3*^*fl/fl*^ and *Uba3*^ΔT^ mice at days 0,5,10 p.i., numbers are density of the bands quantified by scanning densitometry, normalized to GAPDH, relative to that of uninfected *Uba3*^*fl/fl*^ mice. Right, bar graphs showing fold densitometry of indicated molecules in *Uba3*^ΔT^ CD4^+^ T cells compared to that in *Uba3*^*fl/fl*^ counterparts at the same time point (n = 3–4 per group). (D) Left, flow cytometry analysis of mitochondrial membrane potential (ΔΨm, TMRE staining) in splenic CD4^+^ T cells from *Uba3*^*fl/fl*^ (red) and *Uba3*^ΔT^ mice (blue) at days 0,7,11 p.i., cells pretreated with FCCP (+FCCP, black and gray dashed line) to eliminate TMRE signal served as negative controls. Right, bar graphs showing loss of ΔΨm in gated splenic CD4^+^ T cells from naïve and *P*. *yoelii* 17XNL-infected mice (n = 4 per group). (E) Bcl-2 expression in CD4^+^ T cells from *Uba3*^*fl/fl*^ and *Uba3*^ΔT^ mice stimulated with CD3/CD28 Dynabeads for 0, 48 and 60 h. (F) Representative dot plots showing Annexin V and PI staining of CD4^+^ T cells from *Uba3*^*fl/fl*^ and *Uba3*^ΔT^ mice, either prior to (0 h) or after stimulation with CD3/CD28 Dynabeads in the presence of 300 nM ABT737 (+) or DMSO (–) for 60 h. Data are presented as mean±SEM from three or more independent experiments. *p<0.05, ***p<0.01 and ***p<0.001 by Student’s *t* test.

Because apoptotic cell death occurred following TCR activation is another critical determinant of the magnitude of T cell responses [23, 24], we wondered whether neddylation plays a role in the survival/apoptosis of *Plasmodium*-responsive CD4^+^ T cells. For this, apoptotic CD4^+^ T cells presented in the spleen of naïve and *P*. *yoelii* 17XNL-infected mice were assessed by Annexin V and 7-amino-actinomycin D (7AAD) dual staining. Consistent with previous findings [25], CD4^+^ T cell apoptosis was evident around day 7 p.i. and increased progressively with ongoing infection. However, compared to the *Uba3*^*fl/fl*^ littermate controls, *Uba3*^ΔT^ mice displayed higher percentages of apoptotic (Annexin V^+^) CD4^+^ T cells both at day 7 and day 11 p.i. (Fig 7A), suggesting that neddylation may provide an anti-apoptotic signal to CD4^+^ T cells during *Plasmodium* infection. To explore the molecular events, we focused on the signaling of activation induced cell death (AICD) triggered by the death receptors such as Fas [26, 27], and mitochondria-mediated pathway of apoptosis regulated by Bcl-2 family members [28–30]. As analyzed by flow cytometry, there was no difference in Fas or Fas ligand (FasL) expression on splenic CD4^+^ T cells from *P*. *yoelii* 17XNL-infected *Uba3*^*fl/fl*^ and *Uba3*^ΔT^ mice (Fig 7B). However, a significantly lower Bcl-2 expression was found in splenic CD4^+^ T cells from *Uba3*^ΔT^ mice at day 5 and day 10 p.i., although Bcl-X_L_ and Bim levels were similar between the groups (Fig 7C). In line with this, Tetramethylrhodamine ethyl ester (TMRE) staining of the mitochondrial membrane potential (ΔΨm) showed a significant loss of TMRE fluorescence, which is indicative of increased mitochondrial depolarization associated with apoptosis in Uba3-deficient CD4^+^ T cells, as compared with *Uba3*^*fl/fl*^ controls at day 7 and 11 p.i. (Fig 7D). Together, these data support the view that neddylation regulates *Plasmodium*-responsive CD4^+^ T cell survival via a mitochondria-dependent process.

Then we asked whether neddylation exerted such effect directly on CD4^+^ T cells. To this end, naïve CD4^+^ T cells purified from *Uba3*^*fl/fl*^ and *Uba3*^ΔT^ mice were stimulated with CD3/CD28 Dynabeads for 60h prior to detection of cell apoptosis by Annexin V/propidium iodide (PI) staining. In parallel with data from *P*. *yoelii* 17XNL-infected mice, a remarkable Bcl-2 induction was observed upon TCR activation, and the levels were substantially lower in Uba3-deficient CD4^+^ T cells than in Uba3-sufficient controls (Fig 7E). Again, although no difference was found in resting cells, the stimuli led to a 3-fold increase of Annexin V binding in Uba3-dificient CD4^+^ T cells compared with that exhibited in Uba3-sufficient counterparts (Fig 7F). Furthermore, to directly address whether this effect correlated with the Bcl-2 levels, ABT737, a specific antagonist of Bcl-2 was added to the cultures. As anticipated, an obvious increase in the percentage of apoptotic CD4^+^ T cells following stimulation was observed, and the difference between Uba3-sufficient and -deficient cells (30% versus 42%) was markedly decreased, compared to that without ABT737 treatment (10% versus 34%) (Fig 7F). Therefore, neddylation-mediated CD4^+^ T cell survival could be attributed largely to a positive effect on Bcl-2 expression.

Collectively, in addition to augmenting effector CD4^+^ T cell responses, neddylation is a regulator of CD4^+^ T cell survival, mainly through upregulating Bcl-2 and hence suppressing mitochondria-dependent apoptosis during blood-stage *Plasmodium* infection.

### Neddylation contributes to Tfh cell differentiation during *P*. *yoelii* 17XNL infection

**Fig 8:**
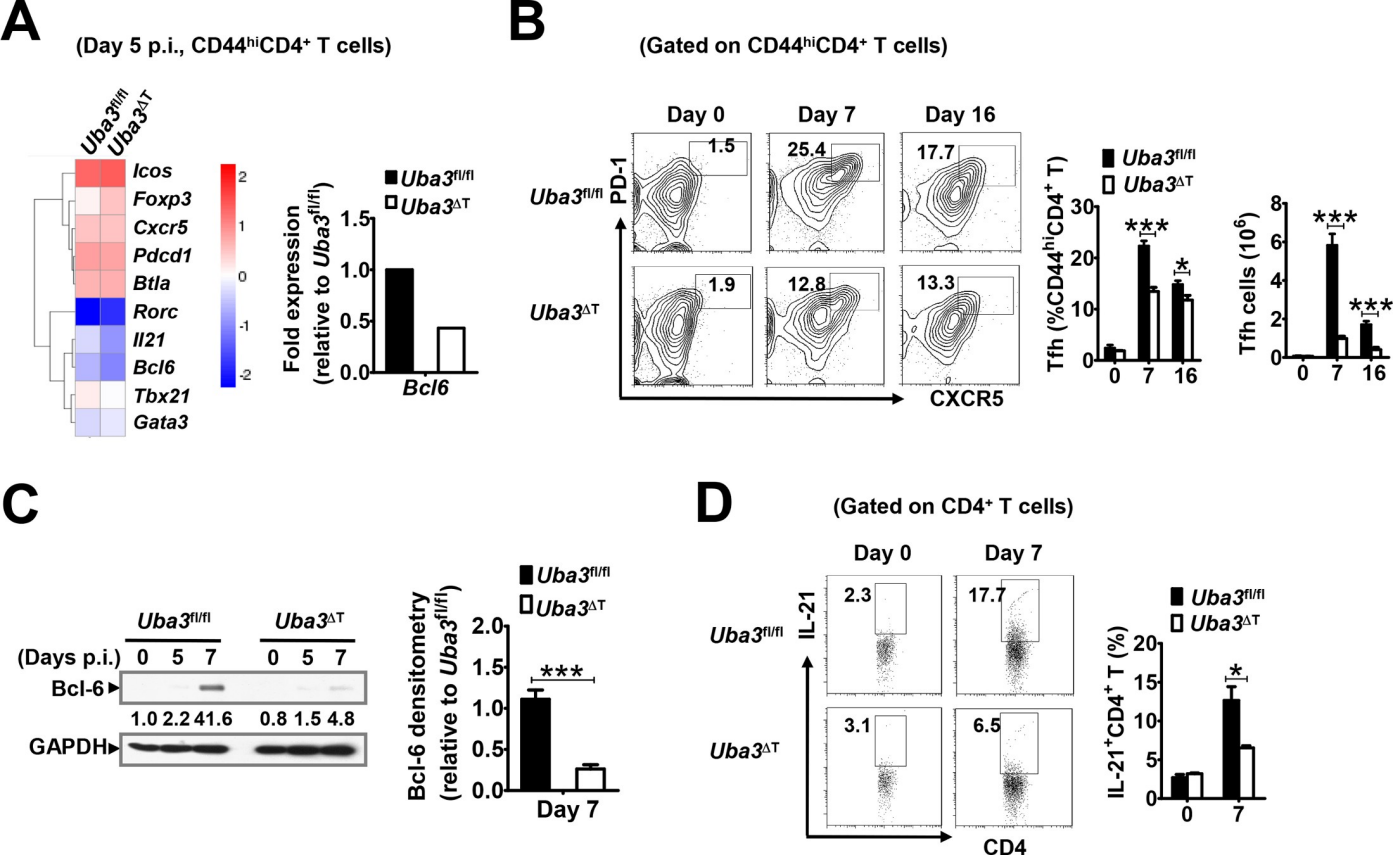
Neddylation contributes to Tfh cell differentiation during blood-stage *P*. *yoelii* 17XNL infection. (A) Left, RNA-Seq analysis of activated CD44^hi^CD4^+^ T cells from 3 mice at day 5 of *P*. *yoelii* 17XNL infection. Heat map showing differential expression of the signature genes related to Th1, Th2, Th17, Tfh and Treg lineages between *Uba3*^*fl/fl*^ and *Uba3*^ΔT^ mice. Right, validation of Bcl-6 expression by quantitative RT-PCR for activated CD4^+^ T cells as described above. (B) Representative counter plots and bar graphs showing the proportions and numbers of Tfh (PD-1^+^CXCR5^+^) cells among activated CD4^+^CD44^hi^ T cells in spleens of *Uba3*^*fl/fl*^ and *Uba3*^ΔT^ mice at days 0, 7 and 16 p.i. (n = 8–9 per group). (C) Immunoblotting for Bcl-6 in splenic CD4^+^ T cells from *Uba3*^*fl/fl*^ and *Uba3*^ΔT^ mice at days 0, 5, 7 p.i. (n = 4 per group), bar graphs showing densitometry of the bands relative to that of *Uba3*^*fl/fl*^ mice. (D) Intracellular staining of IL-21 in splenic CD4^+^ T cells from *Uba3*^*fl/fl*^ and *Uba3*^ΔT^ mice prior to (day 0) and at day 7 p.i. (n = 6 per group). Data are representative of two or more replicate experiments and are shown as mean±SEM. *p<0.05, ***p<0.01 by Student’s *t* test.

Next, we sought to investigate how neddylation in T cells could influence the GC B cell response and, in consequence, parasite-specific IgG production. For this, we performed a RNA-Seq based transcriptome analysis of activated CD44^hi^CD4^+^ T cells from spleens of *Uba3*^*fl/fl*^ versus *Uba3*^ΔT^ mice at day 5 p.i., focusing on the signature genes of different CD4^+^ T cell lineages. We noted that, in addition to a significant reduction in *Tbx21* gene (encoding T-box transcription factor T-bet), which reflected defective Th1-skewed response as described, *Uba3*^ΔT^ mice also exhibited defects in the expression of several follicular helper T (Tfh) cell signature genes, such as *Il21* and *Bcl-6*, the hallmark cytokine and key transcriptional factor of the Tfh lineage [31–34], which was further confirmed by quantitative RT-PCR (Fig 8A). Therefore, we asked whether neddyaltion has an impact on Tfh cells, the key helpers for the GC reaction and T-dependent humoral immunity [34], during the course of *P*. *yoelii* 17XNL infection. To test this, the Tfh phenotype, defined as CXC chemokine receptor 5 (CXCR5) and programmed death 1 (PD-1) co-expression on activated CD44hi CD4^+^ T cells, was examined in *Uba3*^fl/fl^ and *Uba3*^ΔT^ mice by flow cytometry [8, 35]. As expected, a ~40% reduction in the frequency and nearly 80% decrease in the absolute number of CXCR5^+^PD-1^+^ Tfh cells were observed in *Uba3*^ΔT^ mice at day 7 p.i., compared with those in the *Uba3*^*fl/fl*^ controls. A similar phenotype was also found at day 16 p.i., although both the frequency and number of these cells were diminished in the two groups (Fig 8B). Concurrently, Bcl-6 protein level was remarkably lower in Uba3-deficient CD4^+^ T cells than in Uba3-sufficient counterparts at day 7 of infection (Fig 8C), and the proportion of IL-21-producing CD4^+^ T cells in the spleen was also found to be lower in *Uba3*^ΔT^ mice at this time (Fig 8D). Together, neddylation appears to be a positive regulator of Tfh cell development during *P*. *yoelii* 17XNL infection.

### Neddylation promotes Tfh differentiation via regulating Itch targeted FoxO1 degradation

**Fig 9:**
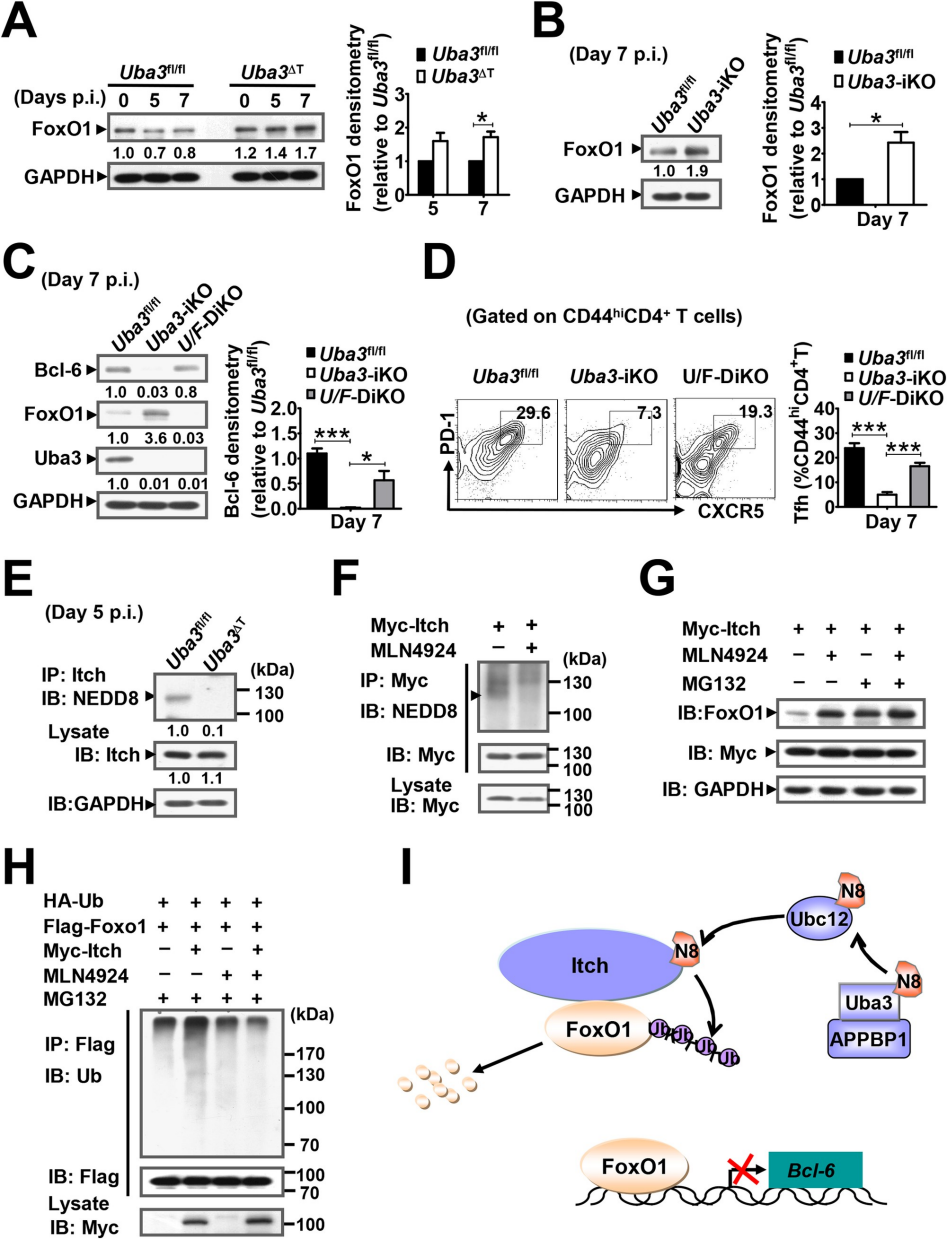
Neddylation promotes Tfh development via regulating Itch targeted FoxO1 degradation. (A) Immunoblotting for FoxO1 in splenic CD4^+^ T cells from *Uba3*^*fl/fl*^ and mice at days 0, 5, 7 p.i. (n = 4 per group), bar graphs showing relative density of FoxO1 in *Uba3*^ΔT^ CD4^+^ T cells compared to that in *Uba3*^*fl/fl*^ counterparts at the same time point. (B) Immunoblotting for FoxO1 in splenic CD4^+^ T cells from *Uba3*^*fl/fl*^ and *Uba3*-iKO mice at day 7 p.i. (n = 3 per group), bar graphs showing relative density of the bands relative to that of *Uba3*^*fl/fl*^ mice. (C-D) *Uba3*^*fl/fl*^, *Uba3*-iKO and *Uba3/Foxo1*-DiKO (*U/F*-DiKO) mice were infected with *P*. *yoelii* 17XNL, Bcl-6 expression and Tfh cell differentiation were determined (n = 4 per group). (C) Immunoblotting for Bcl-6, FoxO1 and Uba3 in splenic CD4^+^ T cells from day 7 infected mice, bar graphs showing densitometry of Bcl-6, relative to that in *Uba3*^*fl/fl*^ mice. (D) Representative counter plots and bar graphs showing the proportions of Tfh (PD-1^+^CXCR5^+^) cells among activated CD4^+^CD44^hi^ T cells in the spleen. (E) Endogenous neddylation of Itch in splenic CD4^+^ T cells from *Uba3*^*fl/fl*^ and *Uba3*^ΔT^ mice at day 5 p.i. was detected by immunoprecipitating (IP) of the cellular lysates with anti-Itch antibody, followed by immunoblotting (IB) with anti-NEDD8 antibody; Lysates without IP were subjected to IB for Itch expression with anti-Itch and anti-GAPDH antibodies. Data represent samples pooled from 3–4 mice per group. (F) 293T cells transfected with Myc-tagged Itch were treated with MLN4924 (2μM) or DMSO (-) for 12 h before collection, neddylated-Itch was detected by IP with anti-Myc antibody, followed by IB with anti-NEDD8 and anti-Myc antibodies. (G) 293T cells transfected with Myc-tagged Itch were treated with MLN4924 or DMSO for 12 h, with or without MG132 (20 μM) being added for the last 8h of culture, FoxO1 and Itch levels was detected by IB with anti-FoxO1 and anti-Myc antibodies, respectively. (H) 293T cells over-expressed with HA-tagged ubiquitin (HA-Ub) and Flag-tagged Foxo1, with or without Myc-tagged Itch cotransfection were treated with MLN4924 or DMSO for 12 h and MG132 for 8 h before collection, ubiquitinated-FoxO1 was detected by IP with anti-Flag antibody, and IB with anti-Ub and anti-Flag antibodies. (I) Schematic representation of Itch as a target in neddylation-promoted Tfh differentiation: NEDD8 modification increases the ubiquitin ligase activity of Itch, leading to degradation of FoxO1 via the ubiquitin-proteasome pathway, which may antagonize the negative role of FoxO1 in Bcl-6 induction, and facilitate the initiation of Tfh cell differentiation. Ub, ubquitin; N8, NEDD8. Data shown in (A-D) are derived from three or more independent experiments. *p<0.05, ***p<0.001 by Student’s *t* test. Data shown in (E-H) are representative of three replicate experiments with similar results.

Then we searched for possible mechanisms underlying the requirement for neddylation in Bcl-6 expression and thus Tfh response. FoxO1, a forkhead-box transcription factor, has recently been identified as an important regulator upstream of Bcl-6 [36]. Despite comparable *Foxo1* mRNA levels (20.86 versus 21.26 by RNA-Seq analysis), immunoblotting analysis revealed considerably accumulated FoxO1 protein in CD4^+^ T cells from *Uba3*^ΔT^ mice, as compared to *Uba3*^*fl/fl*^ mice at day 7 p.i. (Fig 9A). Higher abundance of FoxO1 protein was further confirmed in CD4^+^ T cells from *P*. *yoelii* 17XNL-infected *Uba3*-iKO mice at this time point (Fig 9B). Given that FoxO1 degradation may contribute to Bcl-6 expression and early Tfh differentiation [37], we asked whether the impaired Tfh differentiation correlated with FoxO1 accumulation in Uba3-dificient CD4^+^ T cells. To test this, we polarized naïve CD4^+^ T cells from *Uba3*^*fl/fl*^ and *Uba3*^ΔT^ mice under the condition known to efficiently induce Tfh-like cells, which share fundamental features of Tfh cells [38]. Again, a significant reduction in Bcl-6 mRNA and protein levels, along with substantially higher amount of FoxO1 were found in polarized Uba3-deficient CD4^+^ T cells compared with that in Uba3-sufficient counterparts (S4A Fig). Knockdown of FoxO1 with retrovirus encoding shRNA could largely restored Bcl-6 expression in these polarized Uba3-deifcient CD4^+^ T cells (S4B Fig). Then we determined whether FoxO1 deficiency could rectify the Tfh phenotpe *in vivo*. For this purpose, we crossed *Uba3*^*fl/fl*^*Cd4-Cre*ER^T2^ mice with *Foxo1*^*fl/fl*^ mice to generate *Uba3*^*fl/fl*^*Foxo1*^*fl/fl*^*Cd4-Cre*ER^T2^ mice, in which deficiency of both Uba3 and Foxo1 in peripheral CD4^+^ T cells was achieved by tamoxifen treatment (referred to as *Uba3/Foxo1*-DiKO mice thereafter) (Fig 9C). As expected, mice lacking both Uba3 and FoxO1 in splenic CD4^+^ T cells displayed considerably increased Bcl-6 expression when compared with that in *Uba3*-iKO mice at day 7 p.i. (Fig 9C). Consistent with this, the development of the Tfh population in response to *P*. *yoelii* 17XNL was largely restored in the spleen (Fig 9D). These data suggest that FoxO1 degradation might contribute, at least in part, to neddylation-promoted Bcl-6 induction and hence Tfh expansion during acute *P*. *yoelii* 17XNL infection.

We then explored the relevance of such finding with possible NEDD8 targets. Since the ubiquitin ligase Itch is required for Tfh cell differentiation during acute viral infection, which indeed acts through ubiquitination-mediated degradation of FoxO1 [37], and there is evidence that Itch activity augmented by a mono-neddylation process could facilitate proteosomal degradation of its targets, such as the transcription factor JunB [39]. These findings prompted us to postulate that Itch might provide a link between neddylation and FoxO1 degradation. As expected, we detected endogenous NEDD8 modification of Itch in purified CD4^+^ T cells from *Uba3*^*fl/fl*^ mice at day 5 p.i. that was substantially reduced in CD4^+^ T cells from *Uba3*^ΔT^ mice without disturbing Itch expression (Fig 9E). To better understand whether such modification could influence FoxO1 degradation, we performed verification experiments in HEK293 T cells. Consistent with *P*. *yoelii* 17XNL infection, immunoprecipitates from 293 T cells over-expressed with Myc-tagged Itch confirmed NEDD8 conjugation with Itch, which was almost completely absent by addition of MLN4924 (Fig 9F). Immunoblotting of the cellular lysates from 293T cells over-expressed with Myc-tagged Itch clearly demonstrated that, MLN4924 treatment greatly increased FoxO1 protein, even comparable to the level of that treated with the proteasome inhibitor MG132 (Fig 9G). Additionally, in the presence of MG132, over expression of Itch in 293T cells that co-transfected with HA-tagged ubiquitin together with Flag-tagged *Foxo1* resulted in greatly increased ubiquitinatiated-FoxO1, which could be abrogated by addition of MLN4924 (Fig 9H). Therefore, as shown schematically in Fig 9I, neddylation may represent a mechanism promoting Itch regulated FoxO1 ubiquitination and proteasomal degradation, which may contribute at least partially to Bcl-6 expression and Tfh development during blood-stage *Plasmodium* infection.

## Discussion

*P*. *yoelii* 17XNL provides an ideal tool for comprehensive understanding of T cell immune mechanisms in blood-stage malaria, as both effector T cells and help for antibody responses are implicated in protection [40,41]. In this report, we directly elucidated the biological significance of neddylation in T cell-mediated immunity to *Plasmodium* with this strain. We show that, similar to TCR engagement *in vitro*, *P*. *yoelii* 17XNL can efficiently induce protein neddylation in T cells, abrogation of this process resulted in exacerbated and persistent parasite burdens, leading to a 100% lethality of infection in mice, which seems to be linked to inadequate IFN-γ response and impaired humoral immunity. These findings emphasize a prominent role of neddylation in T cell function [14], and provide evidence for such pathway as a protective mechanism in immune-mediated defense against blood-stage malaria.

CD4^+^ T cells have long been considered as major contributors for protective immunity against blood-stage *Plasmodium* [6, 17]. Our present study highlights the importance of neddylation in proper CD4^+^ T cell responses to *P*. *yoelii* 17XNL infection. Although not shown by Ubc12 knockdown [14], genetic abrogation of neddylation in T cells, targeting either *Uba3* or *Nedd8*, led to a noticeable decrease of mature CD4^+^ T cells in the periphery (Fig 6C and S5 Fig), which might be attributed to a potential role of neddylation in T cell development or homeostatic proliferation and survival. Therefore, it should be taken into account that not only *P*. *yoelii* 17XNL-driven CD4^+^ T cell activities, but also the lower basal level of this cell population, might be responsible for the defects in the magnitude of CD4^+^ T cell responses in these mice. Here, to rule out the possibility that defects in T cell development or homeostasis accounting for the impaired anti-*P*. *yoelii* 17XNL immunity in *Uab3*^ΔT^ and *Nedd8*^ΔT^ mice, we generated *Uba3*-iKO mice, which enabled us to delete *Uba3* in CD4^+^ T cells without affecting cell number under steady state, the finding that the defective anti-*P*. *yoelii* 17XNL immune responses recapitulate those in *Uba3*^ΔT^ mice strongly support the idea that the protective action of neddylation is largely attributed to *Plasmodium*-elicited CD4^+^ T cell functions. The impairment in CD4^+^ T cell expansion and differentiation into Th1 cells may explain considerably to insufficient IFN-γ effector response for parasite control.

As an important mechanism regulating lymphocyte responses and maintaining immune homeostasis, programmed cell death also occurs during malaria infection [25, 42]. Here, we revealed a prosurvival effect of neddylation on *P*. *yoelii* 17XNL-primed CD4^+^ T cells, which was even more pronounced under the condition of TCR ligation *in vitro*, thus implying an autonomous role for this pathway in TCR-induced CD4^+^ T cell survival. The fact that neddylation inhibition accelerated apoptosis of CD4^+^ T cells around the peak of the expansion phase (e.g., day 7 p.i.) might be somewhat correlated with the impaired capability to proliferate in response to *P*. *yoelii* 17XNL infection, as supported by recent data from *P*. *chabaudi* infection [43]. Moreover, among the complex mechanisms involved in T cell survival/death decision, in particular, Fas-mediated extrinsic apoptotic program and mitochondria-dependent intrinsic process represent the major pathways [27, 29]. Consistent with previous research on blood-stage malaria, our data suggest that involvement of neddylation in CD4^+^ T survival does not appear to be linked to Fas-mediated mechanism [44], but rather correlates with the mitochondria associated process, with Bcl-2 being the major molecule to be regulated. As such, improved survival as well as dependence of neddylation on the functional capacity of CD4^+^ T cells may synergistically contribute to an optimal effector response to *Plasmodium* infection.

In addition to Th1 effecor activity, our results highlight a novel role for neddylation in mediating anti-*Plasmodium* humoral immunity, mostly via an effect on Tfh cells. In search for the underlying mechanisms, we demonstrate reduced Bcl-6 accompanied by accumulated FoxO1 proteins in CD4^+^ T cells of *Uba3*^ΔT^ mice, either under the circumstance of *P*. *yoelii* 17XNL infection or the iTfh cultures. As a master regulator for Tfh development, Bcl-6 is regulated by a variety of transcription factors and signaling molecules [32]. FoxO1, a transcription factor that implicated in multiple aspects of T cell functions, has been identified as a regulator directly binds upstream of Bcl-6 [36]. Recent work showed that as a consequence of inducible T cell co-stimulator (ICOS) signaling, inactivation of FoxO1 resulted in a preferential differentiation of Tfh cells. Moreover, genetic deletion of FoxO1 greatly enhanced Bcl-6 expression in T cells [45]. These findings highlighted a negative role for FoxO1 in initiating the Tfh program, even though a positive role in Tfh maintenance has also been identified [45]. Here, the fact that knockdown of *Foxo1* in Tfh-polarized Uba3-deficient CD4^+^ T cells largely rescued the defect in Bcl-6 expression suggests that neddylation-mediated Tfh differentiation, is at least in part, a FoxO1-dependent event. This conclusion was further confirmed *in vivo* by generating a CD4^+^ T cell-specific *Uba3*/*Foxo1* double conditional knockout mice. Furthermore, given the prominent role of neddylation in activating ubiquitin ligases [46], we established a novel link between neddylation and proteasomal degradation of FoxO1, with a particular focus on Itch activity. This finding may provide an explanation for the positive role of neddylation in the development of Tfh and GC reaction, and hence generation of protective antibodies during *P*. *yoelii* 17XNL infection. However, as it is unrealistic that NEDD8 modification of a single protein could account for the overall effects on the Tfh phenotype, additional NEDD8 target(s) [46] might also contribute to neddylation-promoted Tfh differentiation. Furthermore, given the evidence of a positive role of IFN-γ in Bcl-6 expression and Tfh accumulation in lupus development [47], it is reasonable to speculate that apart from a Th1-dependent IgG response [48, 49], *P*. *yoelii* 17XNL elicited IFN-γ response might has additional effect on the differentiation of the Tfh lineage, thus favoring protective humoral immunity to the infection. Further investigations will be needed to explore these issues.

Another substrate of Itch is JunB [39]. Theoretically there should be JunB accumulation upon neddylation blockade. We analyzed JunB expression in splenic CD4^+^ T cells from naïve and *P*. *yoelii* 17XNL-infected mice, as shown in S6 Fig, *P*. *yoelii* 17XNL infection induced a significant increase in JunB expression in splenic CD4^+^ T cells, but the levels were not significantly different between the two groups. It is possible that other factor(s) mediating JunB expression are also regulated by neddylation. Apparently, the IL-2 phenotype in *P*. *yoelii* 17XNL-infected *Uba3*^*fl/fl*^ and *Uba3*^ΔT^ mice was not associated with the Itch target JunB. Neddylation is essential for TCR ligation-induced ERK activation [14], and ERK plays a predominant role in IL-2 expression through various mechanisms [50]. Therefore, defective IL-2 expression upon neddylation blockade should mainly result from impaired ERK activation.

We also explored a longer-term effect of neddylation on the development of parasite-responsive memory CD4^+^ T cells, based on surface expression of CD44, CD127, CD62L [51, 52]. As shown in S7 Fig, we found that both the frequency and number of central memory (defined as CD44^hi^CD127^hi^CD62L^hi^) CD4^+^ T cells were greatly lower in the spleen of *Nedd8*^ΔT^ mice, as compared to *Nedd8*^fl/fl^ mice at day 21 p.i.. Simultaneously, Annexin V staining revealed that more NEDD8-deficient memory cells underwent apoptosis than their NEDD8-sufficient counterparts. These findings deepened our understanding of neddylation in *Plasmodium*-primed CD4^+^ T cell responses and raise the possibility that, in addition to effector T cell-mediated immune responses, neddylation may also play a role in triggering T cell-mediated long lasting protective immunity against parasite re-infection, or vaccine-induced protection against challenge infection.

In conclusion, we have described a crucial role for neddylation in the control of CD4^+^ T cell responses and subsequent humoral immunity to primary blood-stage *Plasmodium* infection. Further investigation on neddylation substrates involved in this process would provide new clues for better control strategies for human malaria, and may have implications for T cell-mediated immunity to other intracellular pathogens.

## Materials and methods

### Ethics statement

All animal work in this study was approved by the Institutional animal care and use committee of Beijing Institute of Basic Medical Sciences (Permit number: AMMS2015-0618), and was performed in strict accordance with the Guide for the Care and Use of Laboratory Animals in Research of the People’s Republic of China. All efforts were made to minimize suffering.

### Mice

Mice harboring *loxP*-flanked *Uba3* or *Nedd8* alleles (*Uba3*^*fl/fl*^ or *Nedd8*^*fl/fl*^) on a C57BL/6 background have been described [53]. *Foxo1*^*fl/fl*^ mice on the C57BL/6 background and mice expressing Cre recombinase under the control of *Lck* promoter or tamoxifen-sensitive Cre ER^T2^ under the control of *Cd4* promoter were purchased from The Jackson Laboratory (Bar Harbor, ME, USA). *Uba3*^*fl/fl*^ and *Nedd8*^*fl/fl*^ mice were crossed with *Lck-Cre* or *Cd4-Cre*ER^T2^ mice to generate *Uba3*^*fl/fl*^*Lck-Cre*^+^ (*Uba3*^ΔT^), *Nedd8*^*fl/fl*^*Lck-Cre*^+^ (*Nedd8*^ΔT^), and *Uba3*^*fl/fl*^*Cd4-Cre*ER^T2^ mice, respectively. *Uba3*^*fl/fl*^*Cd4-Cre*ER^T2^ mice were further crossed with *Foxo1*^*fl/fl*^ mice to generate *Uba3*^*fl/fl*^*Foxo1*^*fl/fl*^*Cd4-Cre*ER^T2^ mice. For inducible and specific deletion of Uba3 (*Uba3*-iKO) or both Uba3 and Foxo1 (*Uba3/Foxo1*-DiKO) in peripheral CD4^+^ T cells, *Uba3*^*fl/fl*^*Cd4-Cre*ER^T2^ or *Uba3*^*fl/fl*^*Foxo1*^*fl/fl*^*Cd4-Cre*ER^T2^ mice were injected intraperitoneally (i.p.) with 2mg tamoxifen daily for consecutive 5 days. These mice were then infected with *P*. *yoelii* 17XNL on the sixth day. The same treated *Uba3*^*fl/fl*^ or *Uba3*^*fl/fl*^*Foxo1*^*fl/fl*^ mice were served as controls, respectively. All mice were bred and maintained under specific pathogen-free conditions and used between 6 to 8 weeks of age.

### Parasite and experimental infection

The cloned line of blood-stage *P*. *yoelii* 17XNL was stored as described [16]. Infection was initiated by i.p. injection of experimental female mice with 3×10^4^ parasitized erythrocytes. Mortality was monitored daily throughout the course of infection. Parasitemia was quantified by microscopic examination of Giemsa-stained thin smears of tail blood.

### Cytokine and parasite-specific antibody ELISAs

Levels of serum IFN-γ, TNF-α, TGF-β and IL-10 were quantified using Ready- SET-Go ELISA Kits (eBioscience). *P*. *yoelii* 17XNL-specific IgG was detected by coating 96-well plates with whole blood-stage plasmodial antigen prepared according to previously published procedures [16], followed by sequential incubation with diluted serum samples and HRP-conjugated goat anti-mouse IgG. 3,3’,5,5’-tetramethylbenzidine was used as substrate and absorbance was read at 450 nm.

### BrdU incorporation assay

To evaluate the proliferative CD4^+^ T cell response *in vivo*, mice were injected i.p. with 1mg BrdU (Sigma-Aldrich) diluted in sterile PBS on the indicated days of infection. 24h later, mice were sacrificed and single cell suspensions of splenocytes were stained for surface markers followed by detection of BrdU incorporation [54]. In brief, cells were fixed over night in 2% paraformaldehyde, permeabilized with 0.01% Triton X-100/1% BSA/PBS on ice, and then treated with DNase at 37 ºC for 30 min. Subsequently, the samples were intracellularly stained with anti-BrdU (Bu20a) and subjected to flow cytometric analysis.

### CD4^+^ T cell purification and culture

Single cell suspensions of splenocytes were prepared as described previously [16]. CD4^+^ T cells were enriched using CD4^+^ T cell isolation kit (negative selection) or anti-CD4 microbeads (positive selection) (Miltenyi Biotec), with the purity routinely around 95%. For *in vitro* proliferation, purified CD4^+^ T cells were prelabeled with 1μM CFSE (Life Technologies) at 37 °C for 20 min, and thoroughly washed prior to culture. Cells were cultured in RPMI1640 medium supplemented with 10% heat-inactivated fetal bovine serum, 100U/ml penicillin, 100 μg/ml streptomycin, 2 mM L-glutamine and 50 μM β-mercaptoethanol in the presence of Mouse T-Activator CD3/CD28 Dynabeads (Life Technologies) at 37 °C for the indicated periods of time, with or without pretreatment with indicated concentrations of MLN4924 (Active BioChem) or vehicle (DMSO, Sigma-Aldrich) at 37 °C for 1 h. In some experiments, 300ng/ml Bcl-2 inhibitor ABT737 (Santa Cruz) was added to the culture. The resulting samples were harvested and processed for immunoblotting or flow cytometric analysis.

### Mitochondrial membrane potential (Δψm) assessment

Δψm was determined by tetramethylrhodamine ethyl ester (TMRE) staining, according to instructions of the mitochondrial membrane potential kit (Abcam). Briefly, CD4^+^ T cells isolated from naïve and infected mice were resuspended in RPMI 1640 medium, and labeled with 250 nM TMRE at 37 °C for 30 min; Cells pretreated with 50 μM Carbonyl cyanide 4-(trifluoromethoxy) phenylhydrazone (FCCP) for 10 min, a potent mitochondrial oxidative phosphorylation uncoupler which induces depolarization of Δψm, before TMRE staining were served as negative controls. After incubation, cells were collected and TMRE intensity of mitochondria was analyzed by flow cytometry.

### Flow cytometry

Splenocytes from naive or infected mice were Fc blocked and stained with appropriate combinations of FITC-, PE-, PerCP/Cy5.5-, APC-, PE/Cy7-, or Brilliant Violet 421-conjugated monoclonal antibodies (eBioscience or Biolegend). CXCR5 staining was performed using biotinylated anti-CXCR5 (BD PharMingen) and PE-labeled streptavidin (BD PharMingen). For intracellular cytokine staining, cells were restimulated with 50 ng/ml Phorbol-12-myristate-13-acetate (PMA, Sigma-Aldrich) plus 500 ng/ml Ionomycin (Sigma-Aldrich) at 37 °C for 5h in the presence of 1 μl/ml brefeldin A (eBioscience). Surface staining and cell permeabilization were then performed, followed by staining with fluorochrome- conjugated anti-IFN-γ (XMG1.2), anti-IL-2 (JES6-5H4), anti-IL-4 (11B11), anti-IL-17A (TC11-18H10.1), or isotype controls (all from eBioscience). IL-21 was detected using recombinant mouse IL-21 receptor/human Fc chimera (R&D Systems) and PE-conjugated anti-human IgG Fc (ebioscience), as previously described [8]. Foxp3 staining was performed using the Foxp3/transcription factor staining kit (eBioscience). For cell apoptosis, surface stained splenocytes or CD4^+^ T cells recovered from culture were incubated in binding buffer with Annexin V and 7-AAD/PI at room temperature for 10 min in the dark (BD PharMingen). All flow cytometric data were acquired on a FACSCalibur (BD Biosciences) and analyzed with FlowJo software (TreeStar).

### Immunofluorescence and confocal microscopy

Freshly isolated spleens were frozen in Tissue-Tek OCT compound (Sakura Finetek), 6 μm serial sections were fixed with ice-cold acetone for 10 min and air-dried. Endogenous biotin was blocked using the streptavidin/biotin blocking kit (Vector Laboratories) followed by staining with a mixture of Alexa Fluor 488-conjugated rat anti-mouse CD3 (17A2, Biolegend), Alexa Fluor 647-conjugated rat anti-mouse IgD (11-26c.2a, Biolegend), and biotinylated peanut agglutinin (PNA, Vector Laboratories) at 4 °C overnight, PNA was then detected by staining with Alexa Fluor 546-conjugated streptavidin (Thermo Fisher Scientific) for 1 h at room temperature. Slides were washed and mounted in anti-fade mounting medium (Vector Laboratories). Images were acquired using a Nikon Ti-A1 confocal microscope and analyzed with the NIS Elements software.

### Retroviral transduction and Tfh-like cell differentiation

The GFP-expressing retroviral vector LMP and pCl-ECO packaging vector were a kind gift from Lin Sun (Tsinghua University, Beijing, China). LMP-shRNA constructs targeted *Foxo1* were generated by cloning the shRNA-containing nucleotides into the LMP vector in accordance with the manufacturer’s instructions (Open Biosystems). LMP empty vector and LMP-shRNA targeted *Foxo1* were transfected separately into phoenix packaging cells, cell-free supernatants containing viral particles were harvested after 48 h of culture. CD4^+^ T cells purified from *Uba3*^ΔT^ mice were activated with CD3/CD28 Dynabeads for 24 h prior to spin infection (1,800 rpm for 90 min at 32 °C) with the retrovirus in the presence of 8 μg/ml polybrene (Sigma-Aldrich), followed by incubation at 37 °C for another 24h. For differentiation of Tfh-like cells, FACS-sorted naïve CD62L^hi^CD44^lo^CD25^-^CD4^+^ T cells or retroviral transduced GFP^+^CD25^-^CD4^+^ T cells were stimulated with CD3/CD28 Dynabeads under the polarizing condition for 5 days: 10 μg/ml anti-IFN-γ (XMG1.2, eBioscience), 10 μg/ml anti-IL-4 (11B11, eBioscience), 20 μg/ml anti-TGF-β (1D11, BioXCell), 20ng/ml IL-6 (Peprotech) and 50ng/ml IL-21 (R&D Systems), according to previously described methods [38, 55]. The shRNA-containing nucleotide sequences: shRNA1, TGCTGTTGACAGTGAGCGCCCATGGACAACAACAGTAAATTAGTGAAGCCACAGATGTAATTTACTGTTGTTGTCCATGGATGCCTACTGCCTCGGA; shRNA2, TGCTGTTGACAGTGAGCGAATGGAGAACCTTCTGGATAATTAGTGAAGCCACAGATGTAATTATCCAGAAGGTTCTCCATGTGCCTACTGCCTCGGA.

### Cell transfection, immunoblotting and immunoprecipitation

The plasmid encoding Myc-tagged *Itch* was provided by Lingqiang Zhang (Institute of Radiation Medicine, Beijing, China), HA tagged-*Ubiquitin* and Flag-tagged *Foxo1* were purchased from Vigene Biosciences. The primary antibodies against NEDD8 (2745s), FoxO1 (C29H4) and Itch (D8Q6D) were purchased from Cell Signaling Technology, antibodies against Uba3 (ab38649), Myc (ab32) and Ubiquitin (ab140601) were from Abcam, antibodies against Bcl-2 (C-2), β-actin and GAPDH were from Santa Cruz, antibody against Bcl-X_L_ was from Proteintech, antibody against Bim was from ABclonal, antibody against Bcl-6 (1G191E/8) was from BD Pharmingen. Cells harvested for immunoblotting analysis were lysed in ice-cold RIPA buffer supplemented with protease/phosphatase inhibitors. Equal amounts of protein were separated with polyacrylamide gels, electrotransferred onto polyvinylidene difluoride membranes (Millipore), and blotted with appropriate primary antibodies at 4 °C overnight, followed by incubation with corresponding Horseradish peroxidase (HRP)-conjugated secondary antibodies (all from Santa Cruz) at room temperature for 1h. Immunoreactive bands were visualized by the ECL Chemiluminescence Kit (GE Healthcare). For immunoprecipitation assay, HEK 293T cells were transfected with Myc-tagged *Itch*, or Flag-tagged *Foxo1* and HA-tagged *Ubquitin* (with or without Myc-tagged *Itch* co-transfection) by lipofectamine 2000 (Life Technologies). Cells were then cultured in the absence or presence of 2 μΜ MLN4924 for 12 h. In some experiments, the proteasome inhibitor MG132 (Sigma-Aldrich) was added at 20 μM for the last 8h of culture, and the cell lysates were incubated with appropriate primary antibodies and Protein A/G-plus agarose (Santa Cruz) at 4 °C for 4h. The resulting immunoprecipitates were then washed and subjected to immunoblotting analysis as described above. Immunoblot densitometry was analyzed using Image J software.

### RNA sequencing and quantitative RT-PCR

Total RNA was extracted with Trizol reagent (Life Technologies) and quantified using Qubit RNA Assay Kit in Qubit 2.0 Flurometer (Life Technologies, CA, USA). A total amount of 1 μg RNA per sample was used as input material for sample preparations. cDNA libraries were generated using NEBNext UltraTM RNA Library Prep Kit (NEB, USA), sequenced on Illumina HiSeq platform and 150 bp paired-end reads were generated. HTSeq v0.6.0 was used to count the reads numbers mapped to each gene, and FPKM (Fragments per Kilobase of transcript sequence per Millions base pairs sequenced) of each gene was calculated. The sequencing data have been deposited in the Gene Expression Omnibus (GEO) database with accession number GSE111066 (https://www.ncbi.nlm.nih.gov/geo/query/acc.cgi?acc=GSE111066). Quantitative PCR was performed with SYBR Green Mix (Toyobo) on a CFX96 Real-Time system (Bio-Rad). Relative expression of target genes was normalized to the *Gapdh* internal control (2^-ΔΔCt^ method). The primer sequences were as follows: *Bcl-6*, 5’-AAATCTGTGGCACTCGCTTCC-3’ and 5’-CGCAGTTGGCTTTTGTGACG-3’; *Gapdh*: 5’-GTGTTCCTACCCCCAATGTGT-3’ and 5’-GTCATACCAGGAAATGAGCTTGA-3’.

### Statistical analysis

Data were analyzed with GraphPad Prism software (version 5). Statistical comparisons were generally performed by Mann-Whitney or two-tailed, Student’s *t* test. Kaplan–Meier curves of overall survival were compared using the log-rank test. A *p* value < 0.05 was considered to be statistically significant.

## Supporting information

S1 Fig Identification of the mouse model of T cell-specific NEDD8 deficiency. Immunoblotting for NEDD8-conjugated cullins and free NEDD8 expression in thymocytes, splenic T cells, and splenic B cells isolated from *Nedd8*^*fl/fl*^ and *Nedd8*^*fl/fl*^*Lck-Cre*^+^ (*Nedd8*^ΔT^) mice. Numbers are densitometry of the bands relative to that of *Nedd8*^*fl/fl*^ mice. Data are representative of three independent experiments with similar results.
(TIF)

S2 Fig Effect of neddylation on IL-2 production and Th2, Th17, Treg cell differentiation. Intracellular staining of (A) CD4^+^ T cell-derived IL-2, (B) IL-4-producing Th2, (C) IL-17A-producing Th17, (D) Foxp3^+^CD25^+^ Treg cells in spleens of *Uba3*^*fl/fl*^ and *Uba3*^ΔT^ mice during the early phase of infection. Representative dot plots and summary graphs showing the proportions of these subsets in gated splenic CD4^+^ T cells (n = 5–6 per group). Data are representative of three replicate experiments and are shown as mean±SEM. *p<0.05, ***p<0.001 by Student’s *t* test.
(TIF)

S3 Fig Quantification of IFN-γ expression in different T lymphocyte subsets during *P*. *yoelii* 17XNL infection. (A-B) Representative dot plots and summary graphs showing the proportions of IFN-γ-producing cells in splenic CD8^+^ T cell and γδ T cell subsets prior to and at day 5 p.i.. (C) Numbers of IFN-γ^+^CD4^+^ T cells, IFN-γ^+^CD8^+^ T cells and IFN-γ^+^ γδ T cells in spleens of *Uba3*^*fl/fl*^ and *Uba3*^ΔT^ mice at day 5 p.i. (D) Numbers of splenic CD8^+^ T cell in uninfected and day 5 infected *Uba3*^*fl/fl*^ and *Uba3*^ΔT^ mice. Data represent 5–6 mice per group from two or more replicate experiments and are shown as mean±SEM. ***p<0.001 by Student’s *t* test.
(TIF)

S4 Fig An involvement of neddylation in FoxO1 regulated Bcl-6 expression under *in vitro* Tfh polarizing conditions. (A) Left, quantitative RT-PCR for Bcl-6 mRNA in naive and Tfh-polarized Uba3-sufficient and Uba3-deficient CD4^+^ T cells. Data shown are relative to the level of naïve Uba3-sufficient CD4^+^ T cells. Right, immunoblotting and densitometry analysis of Bcl-6 and FoxO1 in Tfh-polarized Uba3-sufficient and -deficient CD4^+^ T cells. (B) Left, quantitative RT-PCR for Bcl-6 mRNA in Tfh-polarized Uba3-deficient CD4^+^ T cells retrovirally transduced with LMP empty vector (ctrl) or LMP-containing shRNA targeted *Foxo1* (shRNA1 and shRNA2). Right, immunoblotting and densitometry analysis of Bcl-6 and FoxO1 in Tfh-polarized Uba3-deficient CD4^+^ T cells retrovirally transduced with LMP empty vector (ctrl) or LMP-containing shRNA targeted *Foxo1* (shRNA1 and shRNA2).
(TIF)

S5 Fig CD4^+^ T cell expansion in *Nedd8*^*fl/fl*^ and *Nedd8*^ΔT^ mice during the early phase of *P*. *yoelii* 17XNL infection. Representative dot plots and bar graphs showing the proportions (gated on live lymphocytes) and absolute numbers of CD3^+^CD4^+^ T cells in spleens of *Nedd8*^*fl/fl*^ and *Nedd8*^ΔT^ mice prior to and at day 5 p.i. (n = 5–6 per group). *p<0.05, **p<0.01 by Student’s *t* test.
(TIF)

S6 Fig JunB expression in CD4^+^ T cells during *P*. *yoelii* 17XNL infection. Immunoblotting and densitometry analysis of JunB in splenic CD4^+^ T cells from naïve and *P*. *yoelii* 17XNL-infected mice. Numbers are density of the bands, normalized to GAPDH, relative to that of uninfected *Uba3*^*fl/fl*^ mice. Data are representative of two independent experiments with similar results.
(TIF)

S7 Fig Neddylation plays a potent role in memory CD4^+^ T cell development during *P*. *yoelii* 17XNL infection. (A) Representative counter plots and bar graphs showing the proportions and absolute numbers of CD62L^hi^CD44^hi^CD127^hi^ central memory CD4^+^ T cells (Tcm: gated on CD44^hi^CD127^hi^CD4^+^ T cells) in spleens of *Nedd8*^*fl/fl*^ and *Nedd8*^ΔT^ mice at day 21 p.i.. (B) Apoptosis of Tcm was assessed by AnnexinV/7AAD staining at day 21 p.i.. Representative counter plots and bar graphs showing the proportions of AnnexinV^+^ apoptotic cells in gated Tcm. Data represent 5 mice per group from two independent experiment and are presented as mean±SEM. *p<0.05 by Student’s *t* test.
(TIF)
