# Supplementary figures and images for: Neddylation contributes to CD4+ T cell-mediated protective immunity against blood-stage Plasmodium infection
Source: PLoS Pathog. 2018 Nov 21;14(11):e1007440. doi: 10.1371/journal.ppat.1007440 (PMC6249024; doi:10.1371/journal.ppat.1007440)

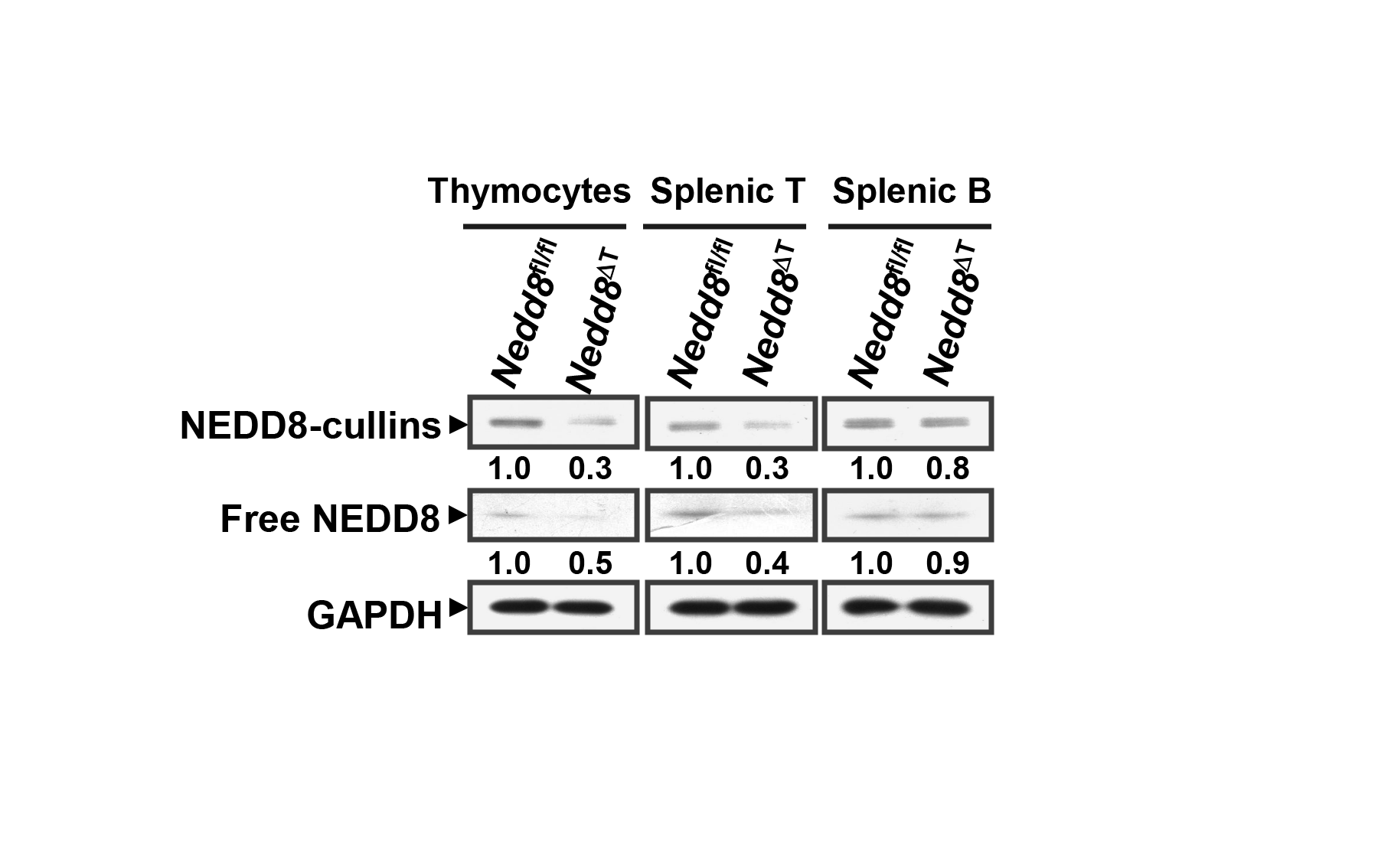

Supplement: S1 Fig — Immunoblotting for NEDD8-conjugated cullins and free NEDD8 expression in thymocytes, splenic T cells, and splenic B cells isolated from Nedd8fl/fl and Nedd8fl/flLck-Cre+ (Nedd8ΔT) mice. Numbers are densitometry of the bands relative to that of Nedd8fl/fl mice. Data are representative of three independent experiments with similar results. (TIF) [file ppat.1007440.s001.tif]

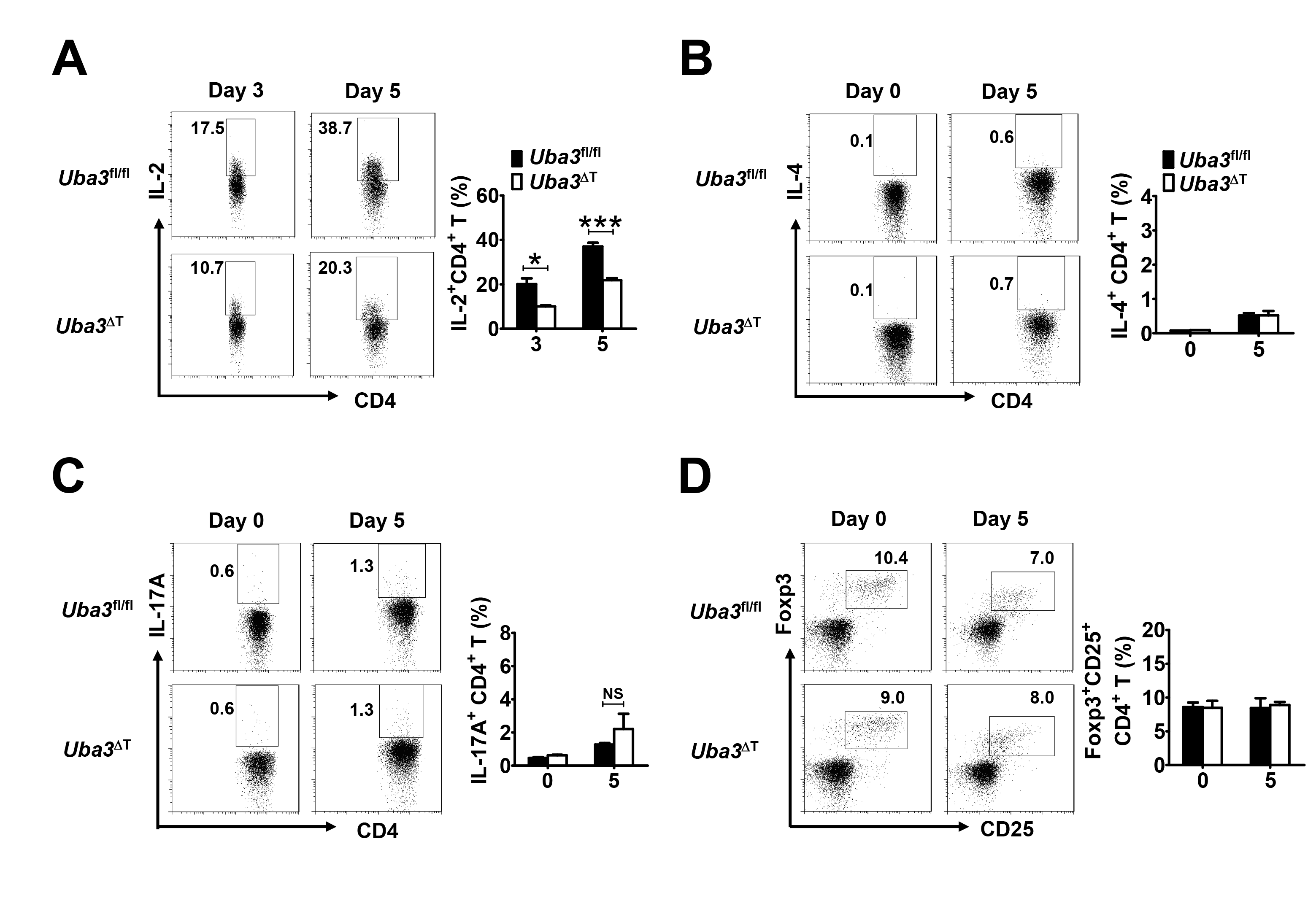

Supplement: S2 Fig — Intracellular staining of (A) CD4+ T cell-derived IL-2, (B) IL-4-producing Th2, (C) IL-17A-producing Th17, (D) Foxp3+CD25+ Treg cells in spleens of Uba3fl/fl and Uba3ΔT mice during the early phase of infection. Representative dot plots and summary graphs showing the proportions of these subsets in gated splenic CD4+ T cells (n = 5–6 per group). Data are representative of three replicate experiments and are shown as mean±SEM. *p<0.05, ***p<0.001 by Student’s t test. (TIF) [file ppat.1007440.s002.tif]

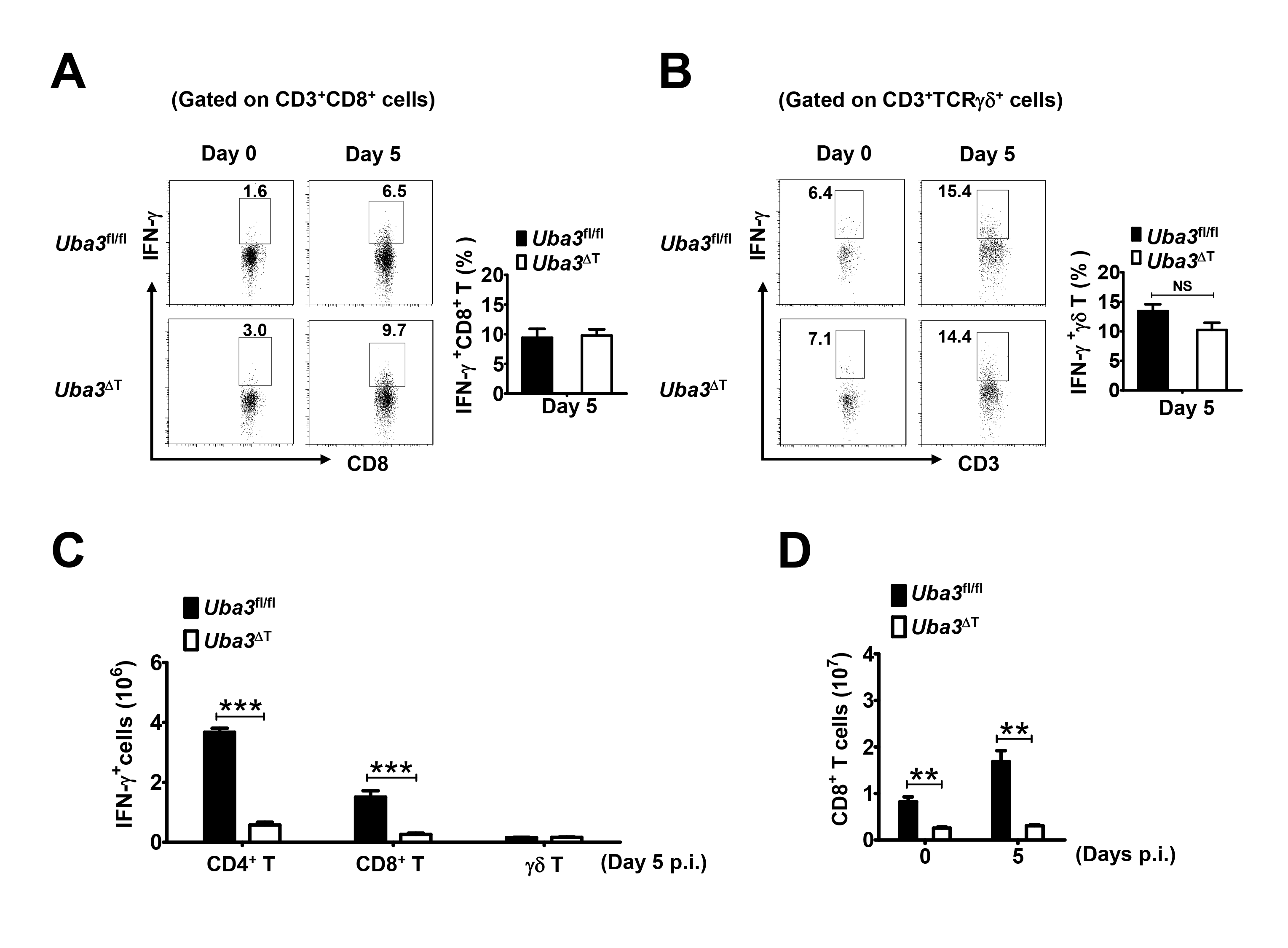

Supplement: S3 Fig — (A-B) Representative dot plots and summary graphs showing the proportions of IFN-γ-producing cells in splenic CD8+ T cell and γδ T cell subsets prior to and at day 5 p.i.. (C) Numbers of IFN-γ+CD4+ T cells, IFN-γ+CD8+ T cells and IFN-γ+ γδ T cells in spleens of Uba3fl/fl and Uba3ΔT mice at day 5 p.i. (D) Numbers of splenic CD8+ T cell in uninfected and day 5 infected Uba3fl/fl and Uba3ΔT mice. Data represent 5–6 mice per group from two or more replicate experiments and are shown as mean±SEM. ***p<0.001 by Student’s t test. (TIF) [file ppat.1007440.s003.tif]

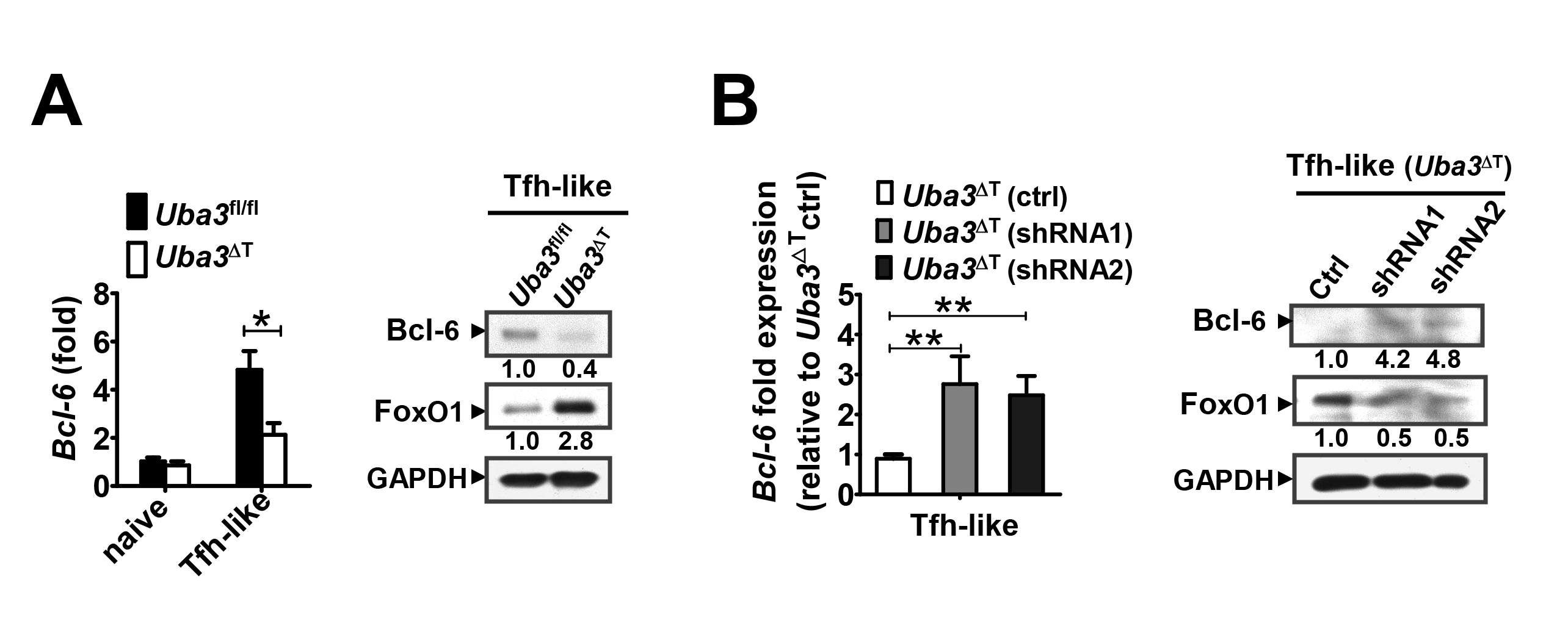

Supplement: S4 Fig — (A) Left, quantitative RT-PCR for Bcl-6 mRNA in naive and Tfh-polarized Uba3-sufficient and Uba3-deficient CD4+ T cells. Data shown are relative to the level of naïve Uba3-sufficient CD4+ T cells. Right, immunoblotting and densitometry analysis of Bcl-6 and FoxO1 in Tfh-polarized Uba3-sufficient and -deficient CD4+ T cells. (B) Left, quantitative RT-PCR for Bcl-6 mRNA in Tfh-polarized Uba3-deficient CD4+ T cells retrovirally transduced with LMP empty vector (ctrl) or LMP-containing shRNA targeted Foxo1 (shRNA1 and shRNA2). Right, immunoblotting and densitometry analysis of Bcl-6 and FoxO1 in Tfh-polarized Uba3-deficient CD4+ T cells retrovirally transduced with LMP empty vector (ctrl) or LMP-containing shRNA targeted Foxo1 (shRNA1 and shRNA2). (TIF) [file ppat.1007440.s004.tif]

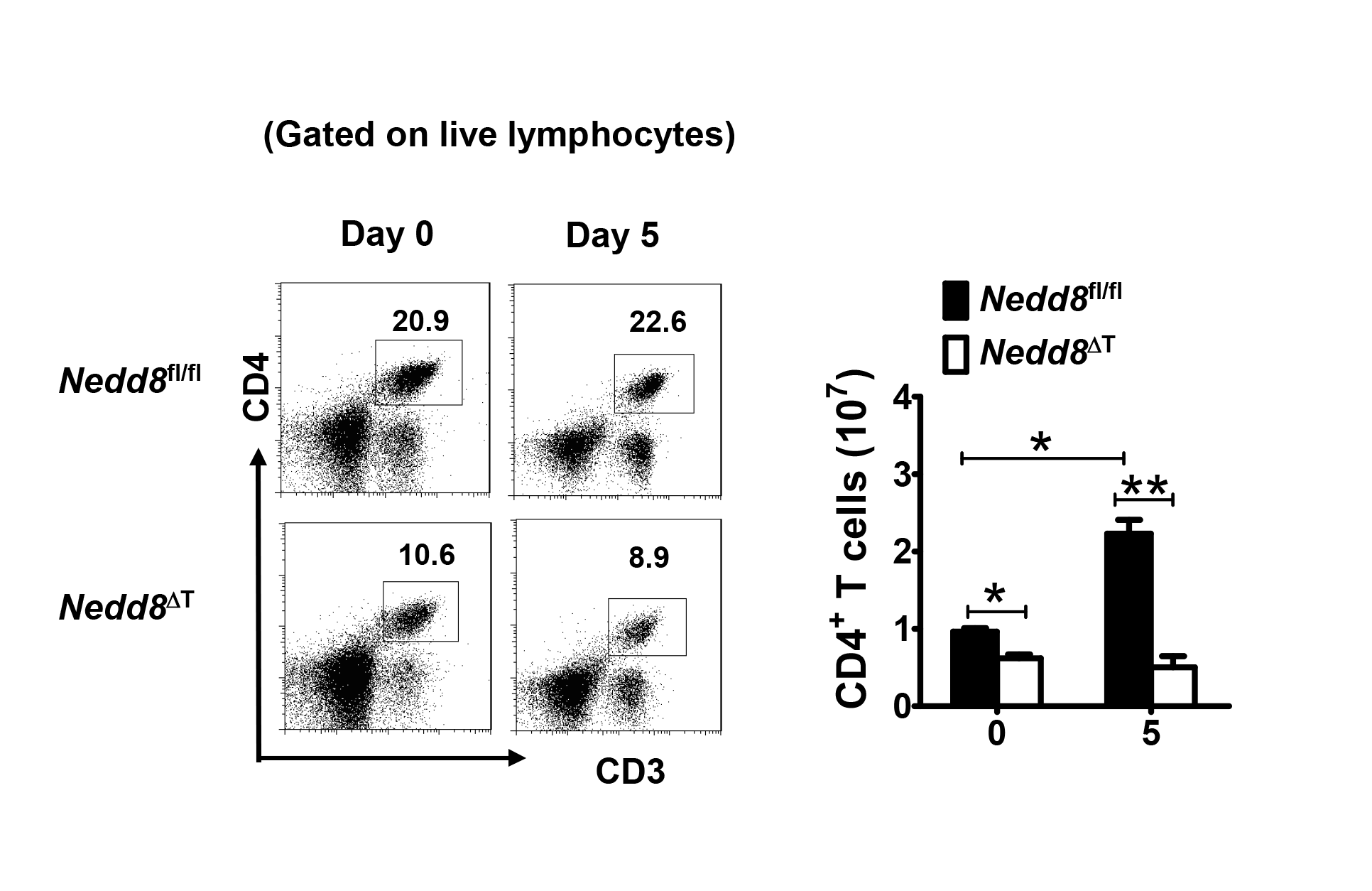

Supplement: S5 Fig — Representative dot plots and bar graphs showing the proportions (gated on live lymphocytes) and absolute numbers of CD3+CD4+ T cells in spleens of Nedd8fl/fl and Nedd8ΔT mice prior to and at day 5 p.i. (n = 5–6 per group). *p<0.05, **p<0.01 by Student’s t test. (TIF) [file ppat.1007440.s005.tif]

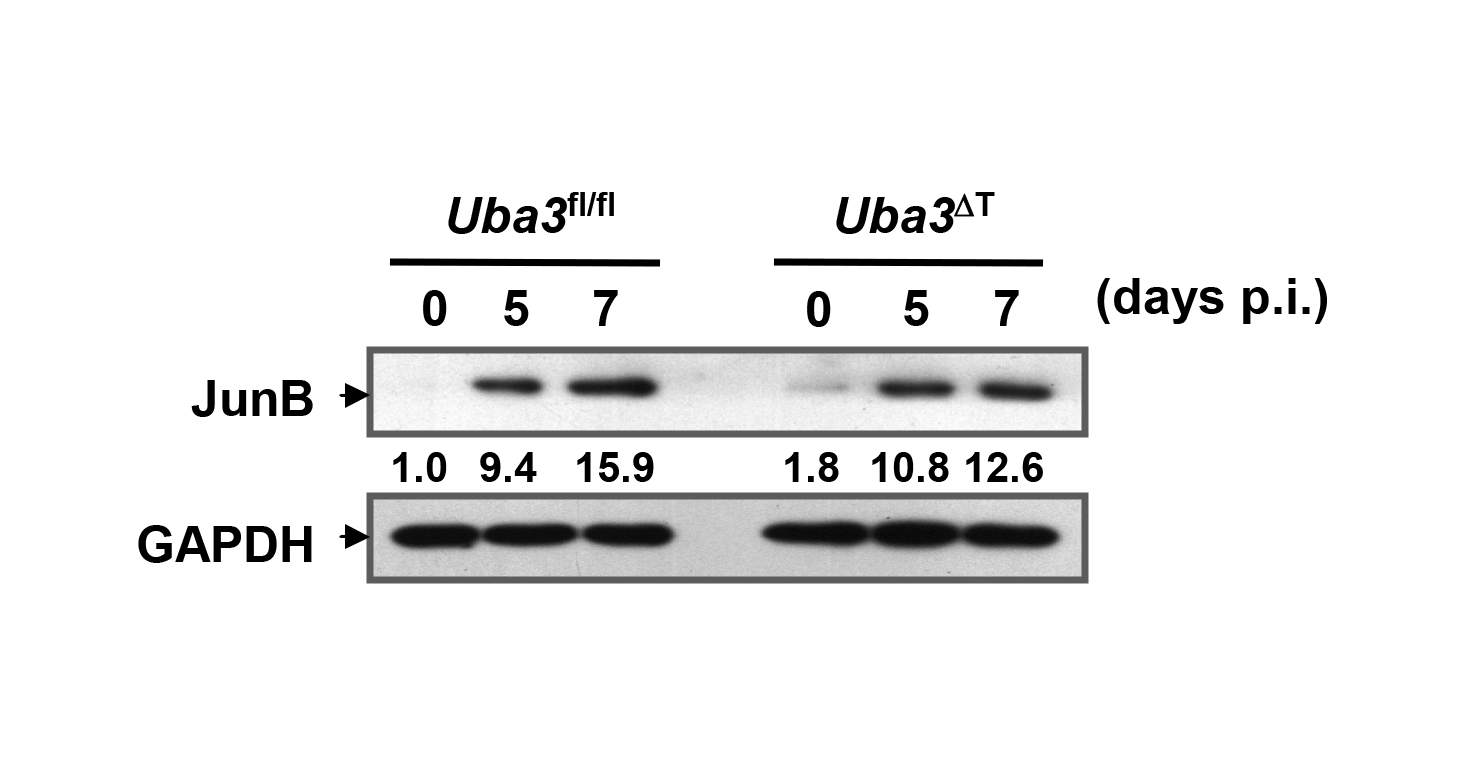

Supplement: S6 Fig — Immunoblotting and densitometry analysis of JunB in splenic CD4+ T cells from naïve and P. yoelii 17XNL-infected mice. Numbers are density of the bands, normalized to GAPDH, relative to that of uninfected Uba3fl/fl mice. Data are representative of two independent experiments with similar results. (TIF) [file ppat.1007440.s006.tif]

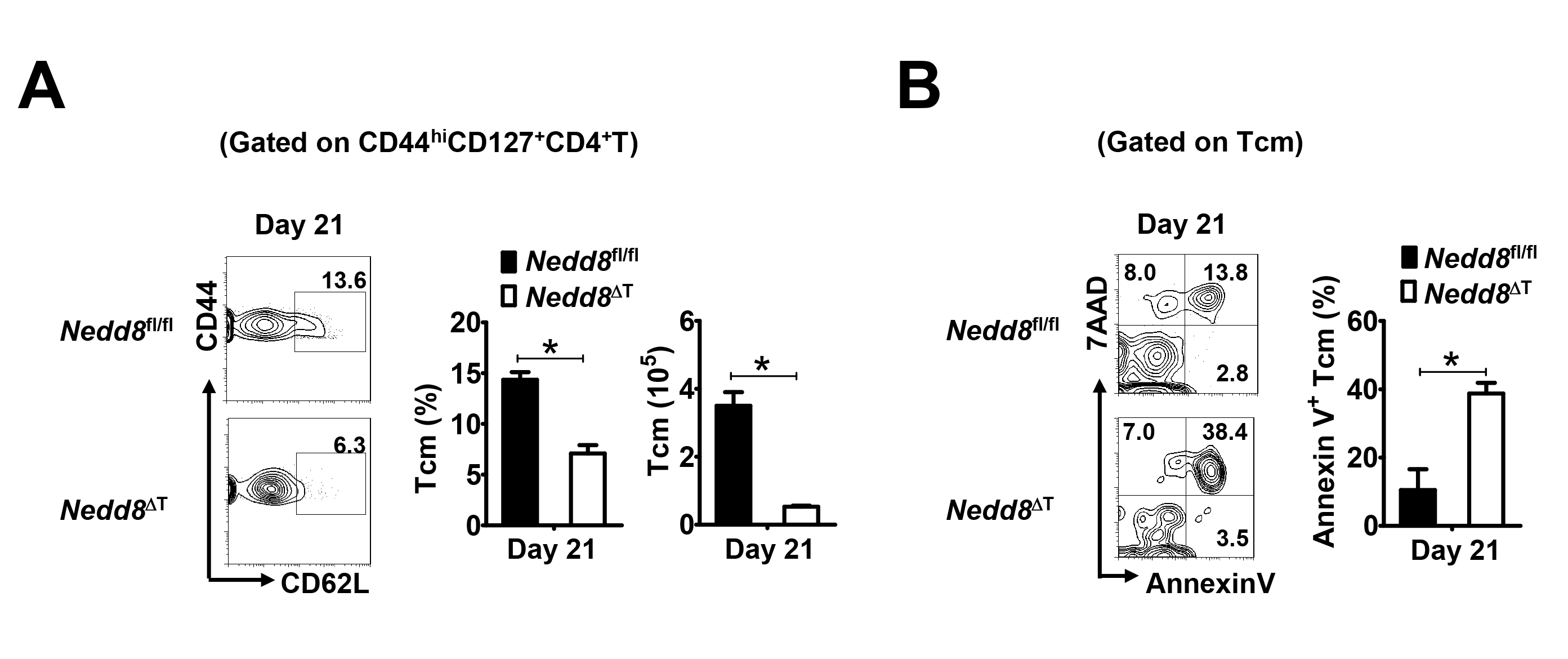

Supplement: S7 Fig — (A) Representative counter plots and bar graphs showing the proportions and absolute numbers of CD62LhiCD44hiCD127hi central memory CD4+ T cells (Tcm: gated on CD44hiCD127hiCD4+ T cells) in spleens of Nedd8fl/fl and Nedd8ΔT mice at day 21 p.i.. (B) Apoptosis of Tcm was assessed by AnnexinV/7AAD staining at day 21 p.i.. Representative counter plots and bar graphs showing the proportions of AnnexinV+ apoptotic cells in gated Tcm. Data represent 5 mice per group from two independent experiment and are presented as mean±SEM. *p<0.05 by Student’s t test. (TIF) [file ppat.1007440.s007.tif]
